# Virtual reconstruction of the Upper Palaeolithic skull from Zlatý Kůň, Czech Republic: Sex assessment and morphological affinity

**DOI:** 10.1371/journal.pone.0201431

**Published:** 2018-08-30

**Authors:** Rebeka Rmoutilová, Pierre Guyomarc’h, Petr Velemínský, Alena Šefčáková, Mathilde Samsel, Frédéric Santos, Bruno Maureille, Jaroslav Brůžek

**Affiliations:** 1 Department of Anthropology and Human Genetics, Faculty of Science, Charles University, Prague, Czech Republic; 2 UMR 5199 PACEA, University of Bordeaux, CNRS, MCC, Pessac, France; 3 Department of Anthropology, National Museum, Prague, Czech Republic; 4 Department of Anthropology, Slovak National Museum-Natural History Museum, Bratislava, Slovak Republic; University of Delaware, UNITED STATES

## Abstract

The incomplete cranium discovered at the Zlatý kůň site in the Bohemian Karst is a rare piece of skeletal evidence of human presence in Central Europe during the Late Glacial period. The relative position of cranial fragments was restored and missing parts of the cranium were virtually reconstructed using mirroring and the Thin-plate splines algorithm. The reconstruction allowed us to collect principal cranial measurements, revise a previous unfounded sex assignment and explore the specimen’s morphological affinity. Visual assessment could not reliably provide a sexual diagnosis, as such methods have been developed on modern populations. Using a population-specific approach developed on cranial measurements collected from the literature on reliably sexed European Upper Palaeolithic specimens, linear discriminant analysis confirmed previous assignment to the female sex. However, caution is necessary with regard to the fact that it was assessed from the skull. The Zlatý kůň specimen clearly falls within the range of Upper Palaeolithic craniometric variation. Despite the shift in cranial variation that accompanied the Last Glacial Maximum (LGM), the Zlatý kůň skull exhibits a morphological affinity with the pre-LGM population. Several interpretations are proposed with regard to the complex population processes that occurred after the LGM in Europe.

## 1 Introduction

The Late Glacial period, characterized by a retreat of the continental ice sheet after the Last Glacial Maximum (LGM; ca 23 to 19 ky cal BP) [1] and abrupt climatic changes [2], provided a rich human skeletal record in Southern and Western Europe. However, there is a paucity of human remains dated to the Late Glacial period in Central Europe [3,4]: an adult skeleton from Bichon in Switzerland (11,760 ± 110 BP) [5], one adult individual from Mittlere Klause in Bavaria (18,590 ± 260 BP) [6], an adult and a newborn from Irlich (11,910 ± 70 BP and 12,110 ± 90 BP) [7], two adult skeletons from Oberkassel in Central Rhineland (11,875 ± 100 BP) [8], the Moča cranium from Slovakia (11,255 ± 80 BP) [9] and a perinatal skeleton from Wilczyce, Poland (12,870 ± 60 BP) [10]. Other localities provided only very fragmentary human remains whose preservation state prevents wider morphological analyses [11–13]. Radiocarbon dating also revealed a more recent age of some specimens previously assigned to the Late Glacial period [11,14]. By contrast, Southwestern Europe excluding Italy had yielded six Middle Magdalenian skeletons and almost 30 specimens dated to the Final Upper Palaeolithic (for a review, see [15–17]).

Here we report on the partially preserved human skull of Zlatý kůň (ZK), one of the rare pieces of skeletal evidence of human habitation in Central Europe during the post-glacial period. Human remains, considered to be from the Early Upper Palaeolithic (EUP) period (see section 2.1.3), were discovered in 1950–53 in a karstic system near the village of Koněprusy in the Czech Republic [18]. During the subsequent fifty years, the Zlatý kůň fossil underwent three major modifications: a reassessment of the number of individuals, a sexual diagnosis and a dating. At first, the remains were considered to belong to more than one individual, one of them being a male [18]. Subsequently, the cranial fragments were assembled together, the number of individuals was reduced to one [19] and the sex of the individual was reevaluated to female [20]. Finally, in 2002, direct radiocarbon dating shifted the fossil from the pre-LGM phase to the post-LGM chrono-cultural period of the Magdalenian [21].

The LGM was a crucial stage in the Late Pleistocene history of the human population. It corresponds to a marked climatic event in the Northern hemisphere [1], characterized by a decrease in temperature and increasing aridity of the environment, leading hunter-gatherers to abandon northern latitudes and to retreat to a southern refuge [22,23]. The LGM also strongly affected the behaviour, morphological features and population genetic structure of European Upper Palaeolithic human groups: Pre- and post-LGM individuals tend to show differences in stature, body proportions and robusticity of the postcranial skeleton as well as differences in cranial morphology [24–28]. In addition, molecular data highlighted a turnover in the composition of European populations correlating with the LGM event and further late-glacial resettlement of Europe [24,25,29,30].

Given the potential significance of the Zlatý kůň skull in the Central European human record, we have undertaken its virtual reconstruction using geometric morphometric techniques. We provide here a brief history of the Zlatý kůň remains, together with photographs and measurements. Considering the new chrono-cultural attribution of the Zlatý kůň specimen and the context of a genetic and morphological shift attributed to the LGM, we addressed two questions concerning the history of the specimen: (1) What is the morphological affinity of the Zlatý kůň skull when compared with extant and UP European populations? and (2) Is it possible to determine the sex of the Zlatý kůň individual based on its cranial morphology? We addressed these questions using linear data extracted from a virtually reconstructed model which we compared with available cranial data obtained from recent and UP specimens. Prospective research directions are proposed which could provide interesting information about population processes in the European Upper Palaeolithic.

## 2 Material

Permission to study the fossil remains of Zlatý kůň (inventory numbers AP2, AP3, AP9, AP10, AP12, AP15, AP17, AP18, AP21) was granted by the Department of Anthropology of the National Museum (Prague, Czech Republic) where the specimen is deposited. The specimen belongs to a publicly accessible collection and was examined with the explicit permission of the appropriate curator (see Acknowledgement). The study was non-invasive and therefore no special permission was necessary. We followed all Czech regulations for fossil studies.

### 2.1 History and preservation of Zlatý kůň

The first skeletal fragments of the Zlatý kůň specimen were discovered in November 1950 in the karstic massif of the Zlatý kůň hill; other skeletal pieces continued to be found until 1953 [18]. The first discovery followed a controlled explosion in a limestone quarry which had made new areas on the second floor of the cave accessible [31]. Many bones of Pleistocene fauna were discovered in the largest part of the cave called Prošek’s Hall, including several fragments of a human posterior calvarium and a right zygomatic bone. These elements were deposited on the surface of a debris cone accumulated through a vertical chimney [32]. The bones were donated to the Geological-Palaeontological Institute of the National Museum in Prague, where they were cleaned and dried [32]. In the spring of 1951, the National Archaeological Institute commenced systematic research on the locality and found more human remains (a left zygomatic bone, a mandible, cervical and thoracic vertebrae and rib fragments), which were accompanied by cultural artefacts [33,34]. The research was methodologically advanced; the team consisted of archaeologists, geologists, palaeontologists and climatologists [35]. The archaeological situation and depositional context are better documented compared to earlier Palaeolithic findings [36]. During the second season in 1952, several vertebral and rib fragments were discovered together with an anterior portion of the calvarium; in 1953 the last piece, a right maxillary fragment, was discovered [18].

Human and other vertebral skeletal remains were found either on the surface of or inside the debris cone. Bones lying on the surface were of a light ochre colour whereas those from inside the debris cone were very dark due to the concentration of manganese in the soil. The latter ones were also reported to be very wet and required a special drying procedure, after which they tended to shrink significantly [32]. The colour difference in particular caused the excavators to believe that two adult individuals were present, designated ZK1 and ZK2 [36,37]. The posterior calvarium, the right zygomatic and the maxilla were assigned to the ZK1 individual; the anterior calvarium together with the left zygomatic and the mandible were assigned to the male specimen ZK2 [18]. However, three decades after its discovery, all fragments were assembled and reinterpreted as only one older adult individual referred to as Zlatý kůň (ZK) [19].

**Fig 1:**
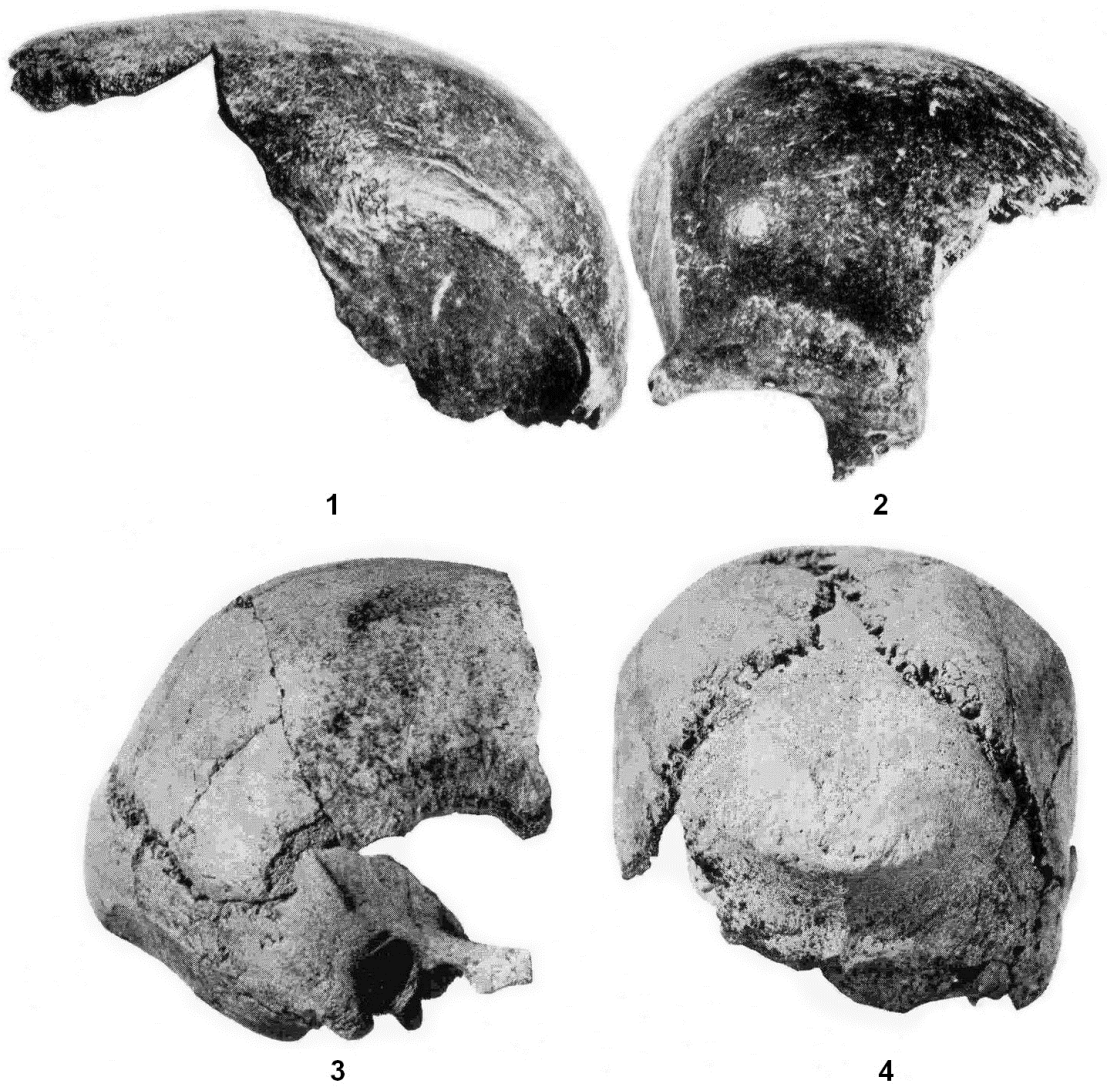
Anterior and posterior (3, 4) cranial portions in the post-recovery state recorded in 1956 (from the Archive of the Department of Anthropology of the National Museum, Prague). Lateral view (1, 3), anterior view (2) and posterior view (4). Not in comparable scale.

The ZK skull is represented by a partial neurocranium and several bones from the facial skeleton. The neurocranial bones were reassembled into two portions of the skull: a posterior and an anterior portion (Fig 1). The posterior portion consisted of fragmentary parietal bones (each assembled from several fragments) and a largely complete occipital bone preserved in one piece with a right temporal bone. After the reassembly, the parietal bones are almost complete, the right one being better preserved than the left. The occipital bone has a very well preserved squama but lacks its basilar part. The temporal bone comprises part of the squama, the zygomatic process, the petrous portion of the temporal bone and the mastoid process. A hole at the intersection of the sagittal and lambdoid sutures indicates an inserted intrasutural bone [34]. The anterior cranial portion consisted of a large frontal fragment with a small parietal portion just posterior to the coronal suture. The left side of the frontal part shows traces of gnawing by a carnivore [38].

The facial skeleton provided incomplete zygomatic bones, the intact mandible and the right maxillary fragment. In contrast to the right one, the left zygomatic bone has a preserved lower orbital margin and a zygomatico-facial foramen. Both zygomatics have clearly visible zygomatico-temporal foramina on the temporal surfaces. The right maxilla is preserved from the level of the second molar to the incomplete alveolar cavity of the first incisor. The frontal process is preserved with the piriform border but the orbital surface and the body are missing. The mandible shows there was an erupted third molar on the left but an unerupted one on the right. All maxillary and mandibular teeth are heavily worn. As documented by Vlček [18], all the teeth were extracted from their dental alveoli and glued back.

**Fig 2:**
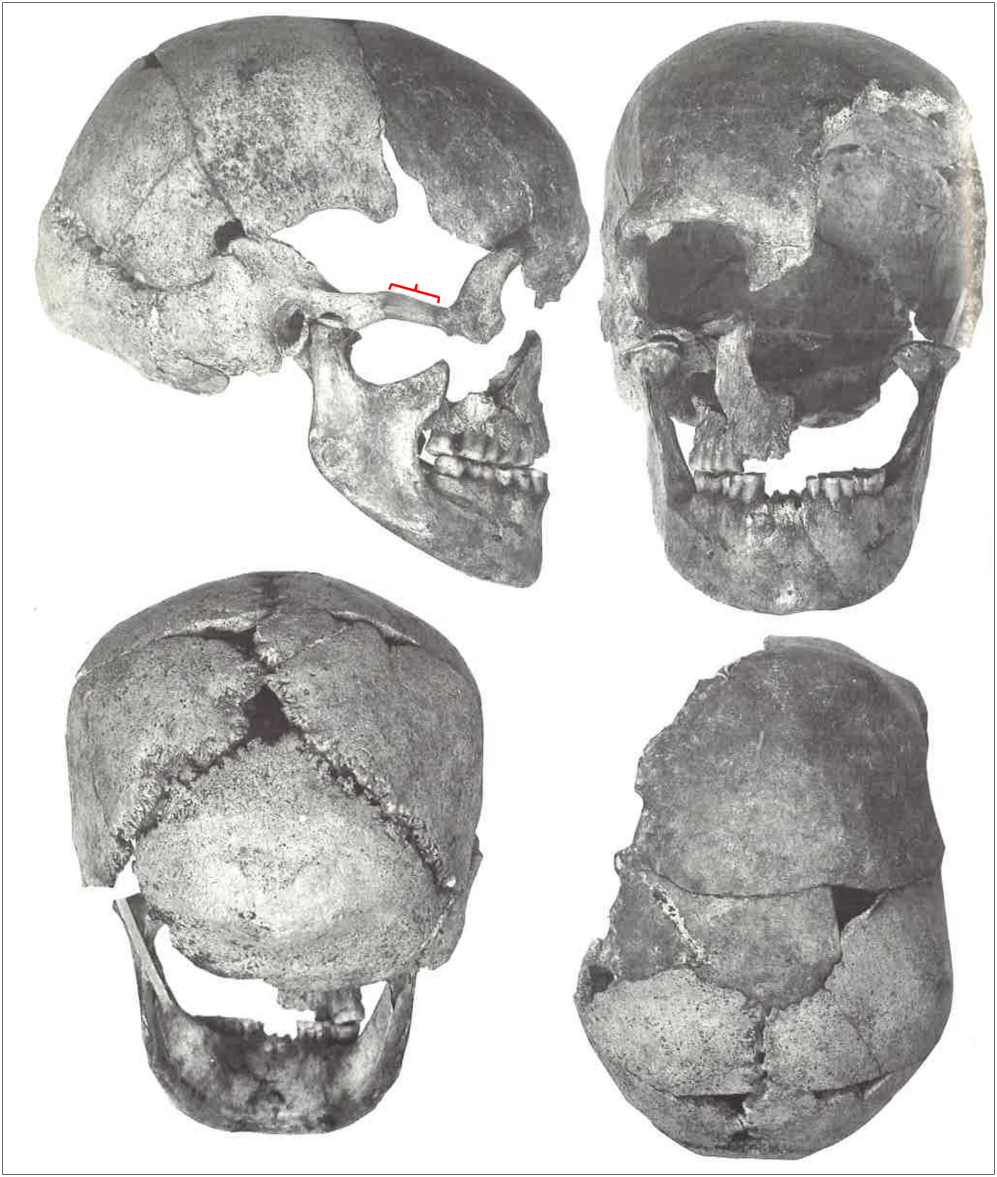
The Zlatý kůň skull with the reconstructed zygomatic arch marked by red curve and attached maxillary fragment (recorded in 1991; from the Archive of the Department of Anthropology of the National Museum, Prague).

**Fig 3:**
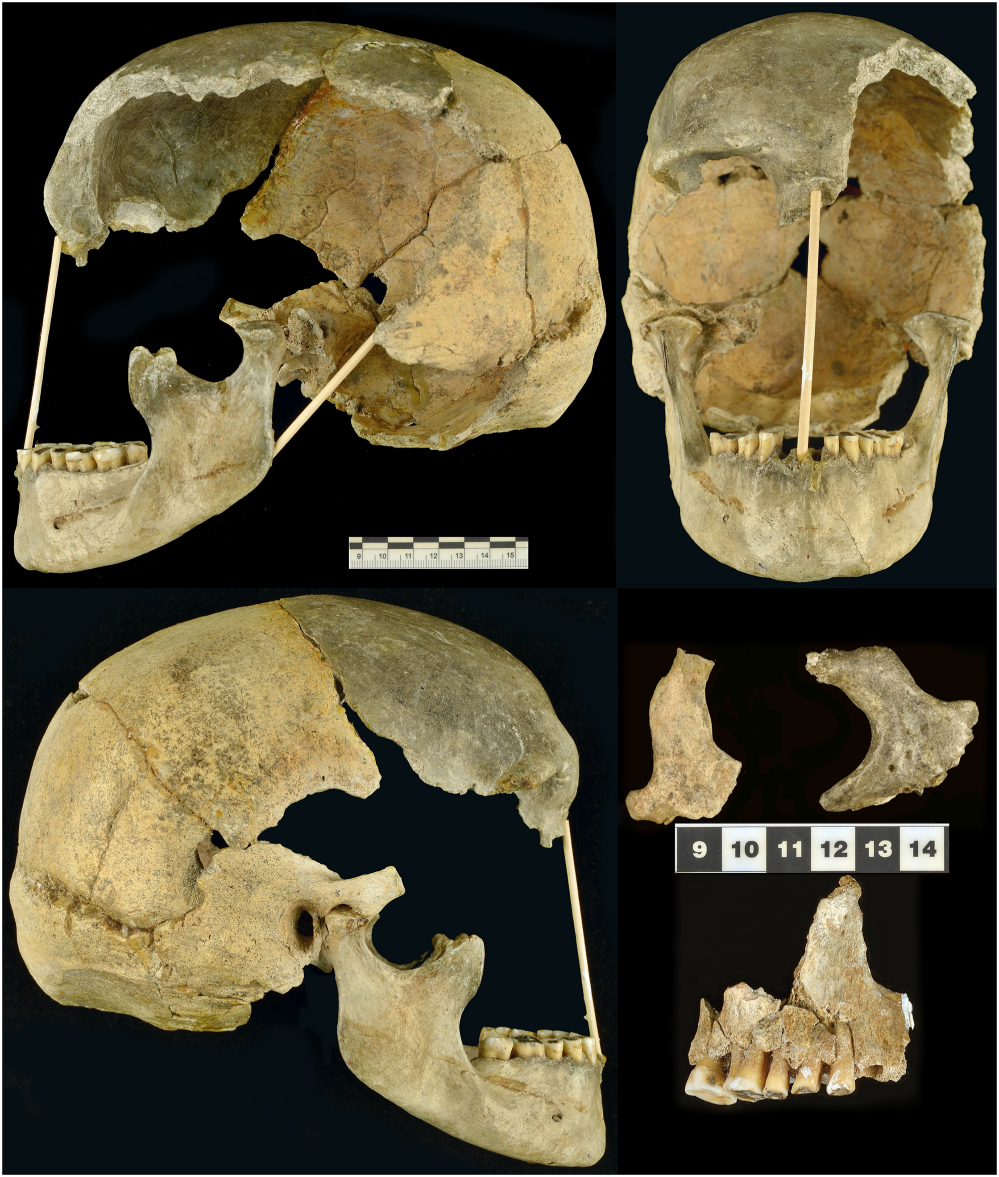
Actual state of the Zlatý kůň skull with separate fragments—Zygomatic bones and maxilla (recorded in 2018; photo Marek Jantač).

The original parts of the Zlatý kůň skull except the left zygomatic bone were manually assembled based on fracture lines and articulations. The re-assembly of fragments also entailed a reconstruction of the missing right zygomatic arch (Fig 2; [20]). However, the absence of the left cranial portion resulted in an anatomically incorrect position of the mandible. The adhesive material did not persist and the formerly attached fragments (the maxilla and the right zygomatic bone) got separated again. Fortunately, the manual modification did not cause any irreversible damage to the fossil. In its present state (Fig 3), only the calvarium and the mandible are still glued together, supported by two wooden sticks; the first from the nasal root to the mandibular alveolus after the right central incisor and the second from the lower edge of the left parietal bone to the left inner mandibular angle.

### 2.2 Previous dating and sex assessment of Zlatý kůň

The first descriptions of the Zlatý kůň specimen indicated that all preserved cranial bones are relatively robust and thick [18,32,33]. Further studies pointed out features similar to Czech EUP (pre-LGM) specimens, namely the occipital bun [19,39], the frontal bone [40] and supraorbital areas, the latter being within the metric variability of specimens from Mladeč, Brno, Dolní Věstonice and Pavlov [41,42]. The cranium’s morphological resemblance to pre-LGM specimens was also emphasized by the statement that “while the geological age and cultural associations of the Zlatý kůň hominid are not entirely certain, the morphological affinities of this specimen are quite clear” [43]. Support for the EUP age was also seen in the depositional context analogous to the Mladeč site, the faunal remains and the assemblage of five artifacts consisting of three lithic tools, a naturally perforated mollusk and a bone point [35,38,44]. However, subsequent direct radiocarbon dating revealed a much younger age close to the end of the Late Pleistocene (12,870 ± 70 uncal BP [21,45]; 15,138–15,635 cal BP, Calib Rev 7.0.4 [46]). Further dating based on a human rib fragment failed to confirm the previous result because of a low amount of ^14^C in the sample [45]. The first dating thus remains the primary evidence concerning the age of the Zlatý kůň cranium.

Many other fossils of presumed EUP age were dated to different periods of the Holocene (Vogelherd [47], Svitávka [21], Svatý Prokop [45], Velika Pećina [48]) and the Zlatý kůň cranium is the only re-dated specimen whose age falls within the Upper Palaeolithic. Chrono-culturally, the specimen is classified to the Magdalenian period of human occupation, archaeologically documented at the nearby sites of Hostim (12,420 ± 470 uncal BP) [49] and Děravá jeskyně [50] but not widely established in Bohemia [51]. This period was characterized by a resettlement of Central and Northern Europe as a consequence of climatic amelioration after the LGM [24]. Nevertheless, Central Europe was still relatively sparsely inhabited in this phase [3].

The two portions of the Zlatý kůň calvarium were thought for a long time to belong to two different individuals (ZK1 and ZK2). The ZK2 individual was considered a male specimen mainly on the basis of sexually dimorphic features in the frontal area—the glabella, supraorbital arch, upper orbital margin and temporal line [18,37,52]. However, further analyses showed that all the cranial fragments match together, representing a single individual [19,20,53]. Since then, the Zlatý kůň skull has been consistently regarded as a female, though without extensive justifications [20,36] and contrary to the previous conclusion [18,19]. The statement was retrospectively supported by Wolpoff et al. [41] based on a morphological comparison with Mladeč 1 and 5 and Pavlov 1 crania; however, sex has been reliably determined only in the case of the last specimen [54]. The poorly preserved postcranial remains of the Zlatý kůň thorax [55] do not provide useful information for the sex diagnosis. As the assessment of sex is also dependent on population variation, we will expand on this topic based upon a comparison of the cranial morphology of the Zlatý kůň individual with that of extant and comparative UP individuals.

## 3 Methods

The virtual reconstruction and subsequent analyses are explained in detail in the following sections. The computer programmes used in each step are listed in section 3.4.

### 3.1 Virtual reconstruction

To virtually reconstruct the skull of Zlatý kůň, the skeletal elements were scanned at the Radiology department of the Hospital Na Homolce in Prague by computed tomography (CT), using a Somatom Sensation 16 scanner (Siemens, Erlangen, Germany). Acquisition parameters were optimally set with a voxel dimension of 0.49 mm, a thickness of 0.6 mm, and a space between slices of 0.3 mm. The wooden sticks were removed from the virtual 3D model and the mandible was separated from the calvarium, as it was misaligned in relation to the midsagittal plane. As a result, we obtained five independent surface models of the calvarium, the right and left zygomatic bones, the right maxilla and the mandible.

**Fig 4:**
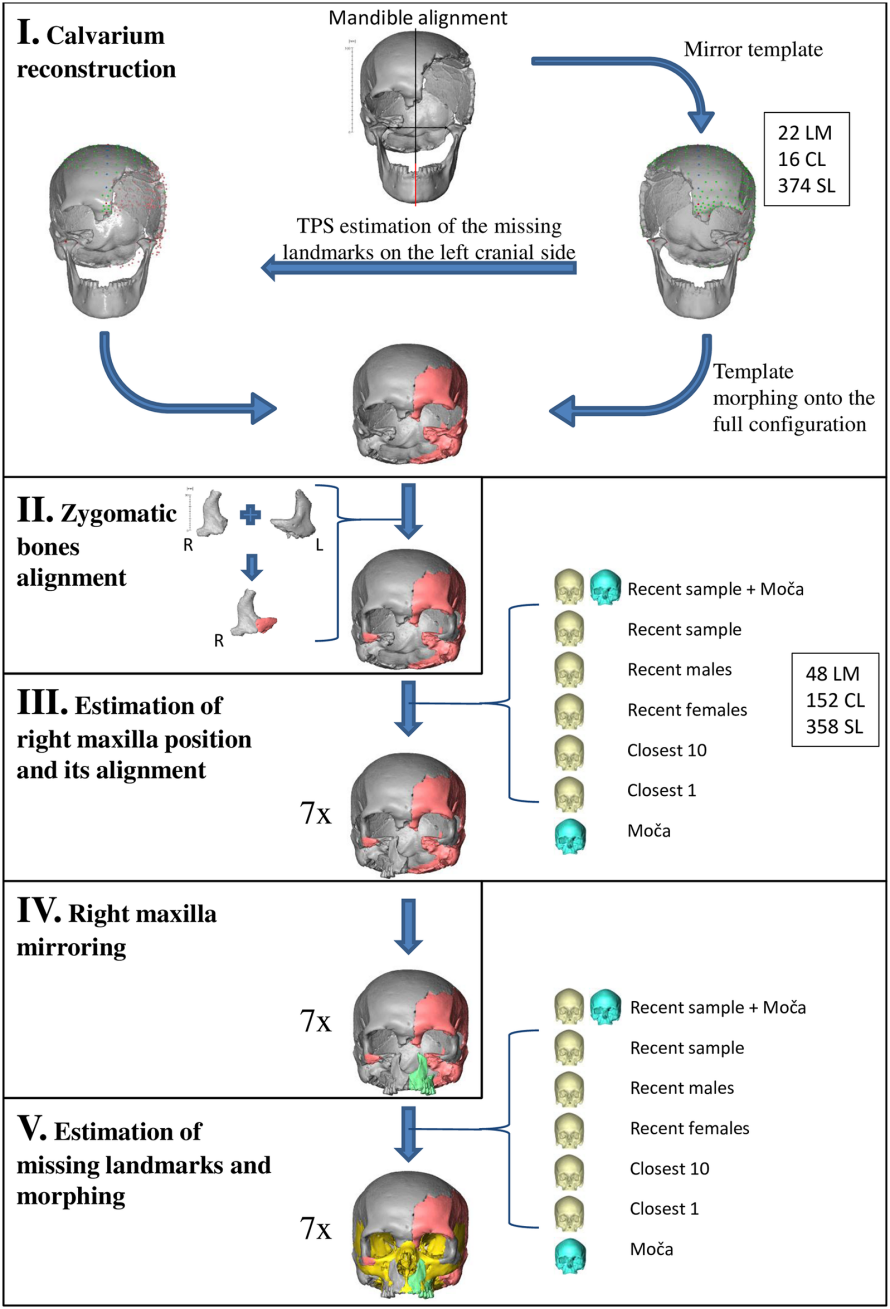
Reconstruction schema. Original bones in grey, preserved-morphology-based reconstruction in orange, mirror-imaging in green, reference-based reconstruction in yellow. Information on number of anatomical landmarks (LM), curve semilandmarks (CL) and surface semilandmarks (SL).

Following the definition of fossil distortions [56], the Zlatý kůň cranium exhibits two types of distortion: fragmentation and missing parts. The main steps of the reconstruction are illustrated in Fig 4, and a list of landmarks and semilandmarks used in each reconstruction step is provided in S1 and S2 Tables. We can divide the reconstruction into two phases: (a) a preserved morphology-based reconstruction and (b) a reference-based reconstruction. In the first phase, we exploited the preserved morphology to reconstruct unilaterally missing parts (steps 1, 2 and 4). In the second phase, we used a reference sample to estimate missing regions (steps 3 and 5). Based on different subsamples derived from the reference sample, we created seven versions of the reconstruction. The reference sample consisted of recent skulls segmented from CT scans acquired at the *CHU de Bordeaux* (15 males, 15 females) and the geo-chronologically closest specimen, the Magdalenian cranium from Moča (Slovakia, ca 13,100 cal BP). The modern crania were used with ethical consent of the *Université de Bordeaux* Institutional Review Board and the *Comité de Protection des Personnes Sud-Ouest et Outre Mer III* (DC 2015/172). The Moča cranium was also CT scanned at the Hospital Na Homolce in Prague, with similar acquisition parameters as those used in the study of the Zlatý kůň specimen.

#### Step 1: Calvarium reconstruction

The calvarium lacks a large portion of its left side, but there is still a large portion preserved posteriorly. Instead of using a simple mirror imaging of the complete right side, we used an approach based on the Thin-Plate Spline (TPS) algorithm to estimate the missing parts of the left side, preserving the natural asymmetry of the cranium [57–59]. As there were not enough anatomical landmarks preserved on the left portion of the calvarium, curve and surface semilandmarks were used. Missing landmarks on the left side were interpolated based on a subset of landmarks available on both sides and the mirror template was then warped to fit the original calvarium.

To obtain a better estimation of the missing contralateral regions, the mandible was attached to the calvarium based on the right temporomandibular articulation and cranial and mandibular midplanes. The midplanes were estimated using Principal component analysis (PCA) of sagittal landmarks [57]. While attached to the calvarium, three translational and two rotational degrees of freedom of the mandible were eliminated (only the rotation along the transversal axis remained), which allowed us to place landmarks on the left mandibular condyle. This procedure was useful in that it preserved the natural asymmetry of the cranium.

#### Step 2: Rearticulation of zygomatic bones

The left zygomatic bone is slightly better preserved than the right one. A mirror image of the left one was therefore used as a template for the completion of the right one in the same way as in the reconstruction of the calvarium. A small portion of the lower orbital margin was thus added to the right zygomatic. Both zygomatic bones were subsequently manually attached to the calvarium based on the direct anatomical connection with the frontal bone and the continuity with the zygomatic process of the temporal bone. This yielded a model of the rearticulated incomplete cranium.

#### Step 3 and 4: Right maxilla rearticulation and mirroring

**Fig 5:**
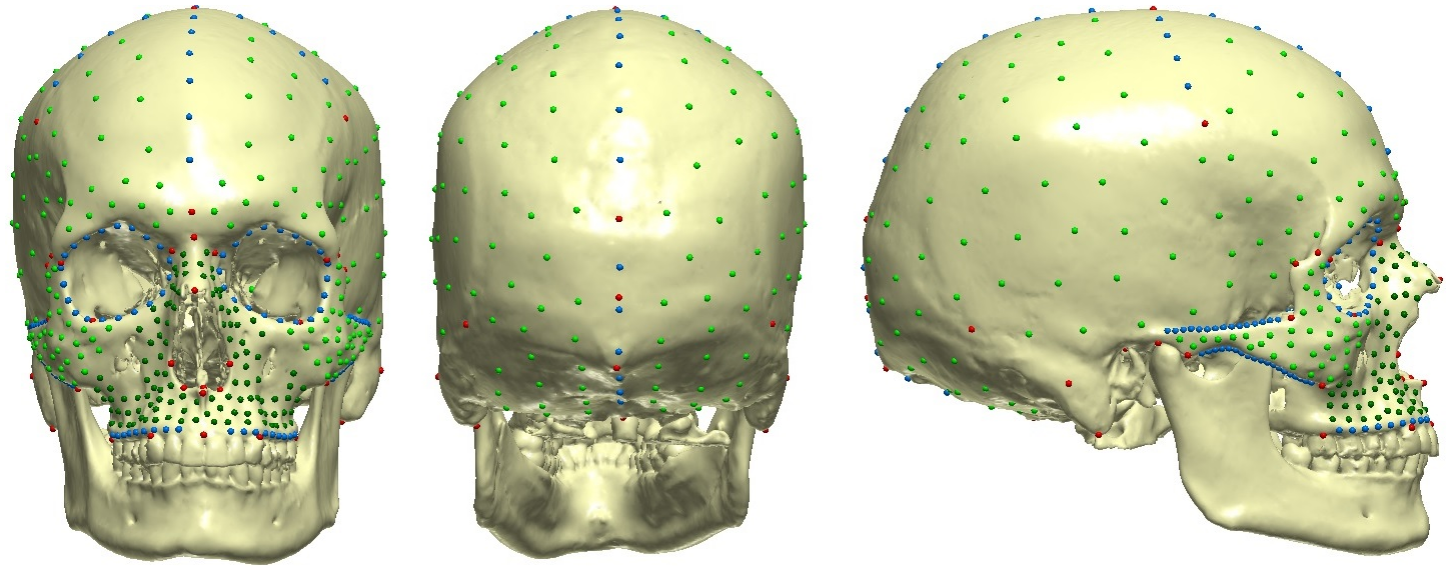
Illustration of landmarks used for the reconstruction. Red (anatomical landmarks), blue (curve semilandmarks), green (surface semilandmarks).

The fragment of the right maxilla could not be directly attached to the cranium, so its position was estimated on the basis of the reference sample of complete crania. The set of landmarks and semilandmarks employed in the reconstruction was digitized on the reference crania and the Zlatý kůň cranium using a predefined template (Fig 5, S1 Table). Maxillary landmarks were estimated relative to the Zlatý kůň cranium based on the different reference subsamples and the maxillary fragment was subsequently aligned into its estimated anatomical position. The estimation was done seven times based on a different selection of reference specimens (Table 1). This resulted in seven models of the cranium with the maxilla attached. In all seven models of the Zlatý kůň skull, the right maxilla was mirrored across the cranial midplane.

**Table 1:** Reference samples for the cranial reconstruction.

Subsample	Description	Number of specimens
1	All specimens (recent crania and Moča)	31
2	Recent specimens	30
3	Recent males	15
4	Recent females	15
5	Closest 10 recent specimens*	10
6	Closest 1 recent specimen*	1
7	Moča	1

*Closest specimens to the incomplete landmark configuration of Zlatý kůň based on Procrustes distance.

#### Step 5: Estimation of missing landmarks and warping

Having all the cranial fragments (excluding the mandible) articulated in their anatomical positions, the remaining missing landmarks were estimated in each of the seven models of the cranium using the same reference sample as in step 3. Finally, the closest complete specimen (according to Procrustes distance) was selected to be warped onto the completed configurations. Areas missing from the Zlatý kůň skull were filled using this warped specimen.

#### Validation

To validate the reconstruction process, a PCA was performed on the full landmark configurations of all the reconstructions and the individuals from the reference sample. The distribution of the reconstructions across the sample allowed us to assess the reliability of the reconstruction process [56]. A Procrustes ANOVA further quantified the variations of the reconstruction procedures compared to the general cranial morphology of the reference sample [60]. Several cranial measurements were also compared to estimated values given by Vlček [18].

### 3.2 Visual sex attribution

As morphometric methods of sex assessment are highly population-specific, a morphoscopic assessment was carried out on the original specimen. Four methods were used to estimate the sex from the cranium and mandible: (a) the traditional method following the recommendations made by the Workshop of European anthropologists evaluating cranial and mandibular characters [61,62]; (b) the method of Walker [63] involving discriminant function analysis (DFA) on five cranial and mandibular features; (c) modified Walker’s method supplemented with scoring of zygomatic extension using decision trees [64]; and (d) a method evaluating the shape of the mandibular ramus [65,66].

### 3.3 Morphometric analysis of affinity and sex attribution

Classical cranial measurements defined by Martin [67] or Bräuer [68] were acquired on the Zlatý kůň reconstructed cranium and analysed in comparison with recent and Upper Palaeolithic European specimens. Three European populations (Austrian, Hungarian and Norse) from the Howells craniometric database (https://web.utk.edu/~auerbach/HOWL.htm) [69,70] were used to represent recent variability of modern humans. Cranial metric data were collected from the published literature on 68 reliably dated UP specimens (for references see S3 Table). Because fossils were often sexed by assessing the skull or the overall robusticity of the skeleton, attention was paid to how their sex was assessed. Information on the sex of individuals was used if it was determined by primary diagnosis from the pelvis [71,72], secondary diagnosis following reliable criteria (see [15,73]) or genetic analysis. Otherwise, the sex was considered unknown.

In total, 20 cranial measurements were inspected (see Results for definitions). From the set of cranial variables, 13 were comparable with variables in the Howells database. Because of the differential taphonomic preservation of the comparative specimens, missing data were imputed using the PCA algorithm, which takes into account similarity between individuals and relationships between variables [74]. On the basis of multiple imputations, we removed specimens for which the presence of missing values caused too much uncertainty about their position along the first two principal axes. This treatment is consistent with the study of Arbour and Brown [75], which concludes that the estimation of missing data provides a better image of reality when only the most uncertain specimens are excluded.

To analyse morphological affinity without the confounding effect of size sexual dimorphism [27], linear measurements were transformed to size-adjusted shape variables via division by the geometric mean and logarithmization [76,77]. UP individuals were divided into two groups, one from before and the other from after the LGM (see [27]). Linear discriminant analysis (LDA) was performed to assess the cranial shape of Zlatý kůň in relation to the different population samples.

To analyse the sex attribution of the Zlatý kůň specimen, raw cranial measurements were used, as size is an important factor in human sexual dimorphism. First, the random forest classification method [78] was applied to the UP sample of known sex. This method usually does not provide high posterior probabilities but allows us to select the most important variables related to sex classification. Subsequently, this set of variables was used in LDA to assign the sex of Zlatý kůň.

### 3.4 Software

The software TIVMI (http://projets.pacea.u-bordeaux.fr/TIVMI) [79] was used to segment surface models from CT scans and to measure linear dimensions on the Zlatý kůň cranium. Surface models were reconstructed from CT scans using the HMH algorithm implemented in TIVMI [80,81]. Landmarks and curve and surface semilandmarks were digitized in Viewbox 4 (dHAL software, Athens, Greece). Semilandmarks were placed semi-automatically using a predefined cranial template. Homologous position of semilandmarks was ensured by a sliding procedure. For more details about sliding semilandmarks in three dimensions see [82]. Missing point estimation, warping and mesh alignment were done using the R package *Morpho* [83]. Surface model processing was done in Meshlab (Visual Computing Lab, Italian National Research Council) and Avizo 7.1 (Visualization Sciences Group, SAS). Data processing and statistical analyses were performed in R 3.3.3 (R Foundation for Statistical Computing, Vienna, Austria, 2015). Missing measurements in the comparative sample were estimated using PCA implemented in the R package *missMDA* [84]. Classification was carried out using the packages *randomForest* [78] and *MASS* [85].

## 4 Results

### 4.1 Reconstruction

The reconstruction of the calvarium resulted in a perfect fit between the reconstructed and preserved morphology that would not be possible with simple mirror imaging because of the presence of natural asymmetry. The smooth transition between the preserved and reconstructed morphology provides confidence that the original morphology of the calvarium is closely approximated.

**Fig 6:**
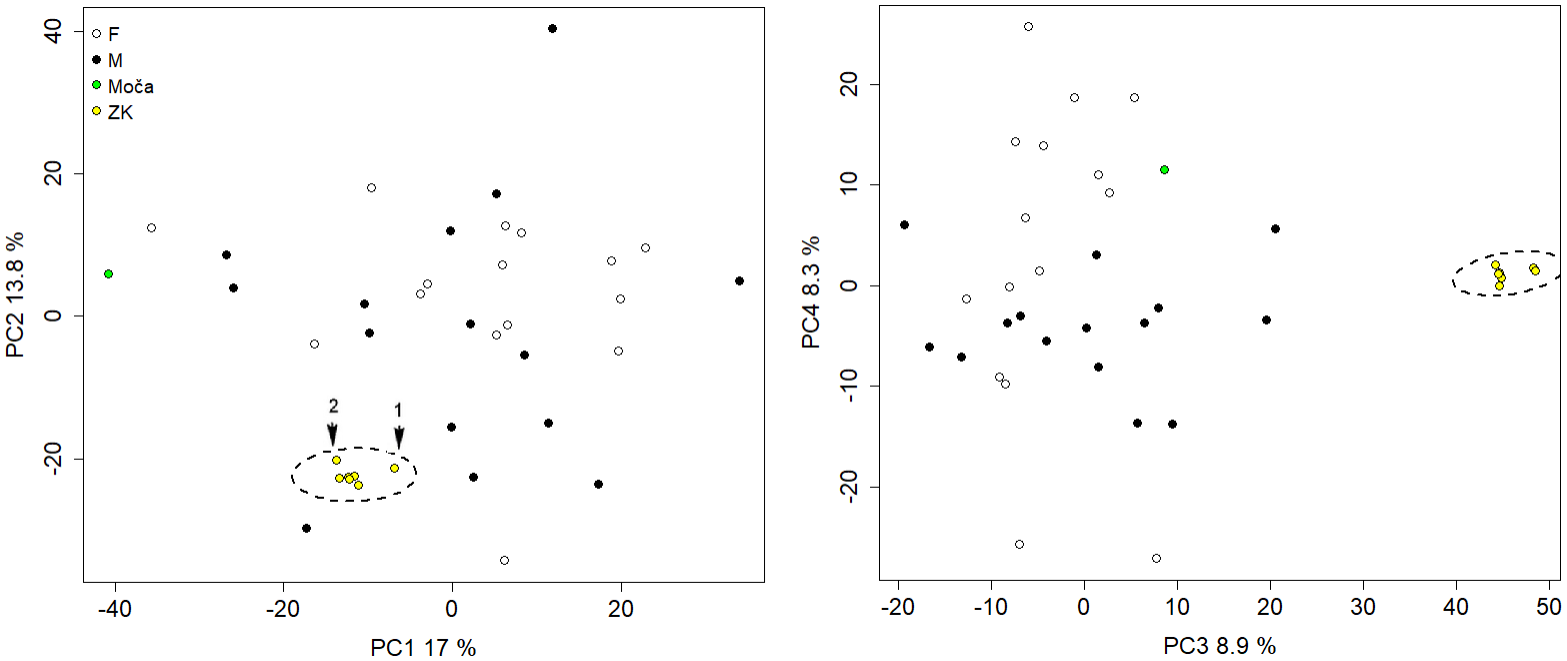
Principal component analysis performed on coordinates of the Zlatý kůň cranial reconstructions and the reference cranial sample. Variability of reconstructions is indicated by 95% confidence ellipses. The most distant reconstructions: 1) based on the closest one specimen, 2) based on the Moča cranium.

**Fig 7:**
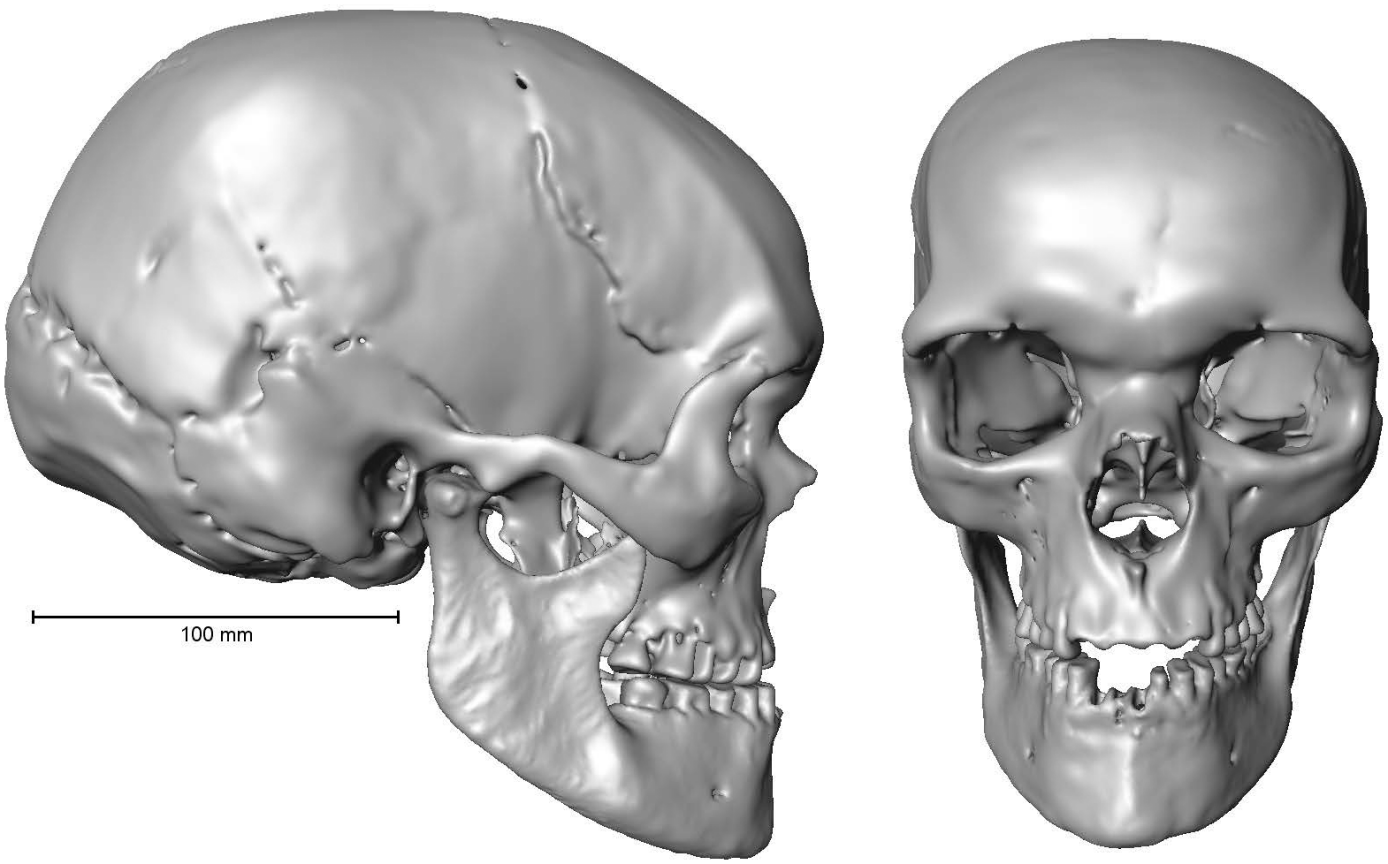
The reconstructed cranium with attached mandible (based on all reference specimens).

In total, seven independent reconstructions of the Zlatý kůň cranium were created based on different reference subsamples used to estimate the position of the right maxilla and the unpreserved regions of the Zlatý kůň cranium. Plotted with other individuals from the reference sample following a PCA on residuals of the Procrustes analysis, the different versions of the reconstructed Zlatý kůň cranium form a cluster with small variation relative to the inter-individual variability (Fig 6), which indicates that there is low uncertainty in the reconstruction. The most different reconstructions of the Zlatý kůň cranium were those based on Moča and the closest specimen (Fig 6). The main difference resided in the position of the maxilla manifesting in maxillary prognathism and maxillary breadth. Both were greater in the Moča-based reconstruction than in the reconstruction based on the closest specimen. The latter was at the same time slightly more distant from all other reconstructions (Fig 6). After the alignment of the mandible (rotation along the axis at the temporomandibular joints), the maxillae did not fit very well in the occlusal plane in both distant reconstructions. On the other hand, in the reconstruction based on the entire reference sample, the maxilla was similarly situated as in Moča but slightly dorso-superiorly rotated, which resulted in better fit with the mandible (Fig 7).

Furthermore, the variation of different reconstructions was quantified using Procrustes ANOVA and accounted for only 2.6% of the total shape variation of the sample and 0.3% in terms of centroid size. Given the small differences between the reconstructions, the one based on all reference specimens was chosen for further morphometric analyses (Fig 7, S1 File).

Several measurements were also taken for comparison with the previous estimates [18]. Depending on the type of reconstruction, they were taken only once (mirroring and preserved morphology-based reconstruction) or from every reconstruction (reference-based reconstruction). Greater differences were noticed in minimum and maximum frontal breadth, spinoalveolar height and nasal breadth, while minimal differences were in palatal breadths (Table 2).

**Table 2:** Comparison of measurements recorded for the Zlatý kůň cranium.

Measurement	M	Vlček [18]	Virtual reconstruction	
Minimum frontal breadth	9	109*	97.2	[Rm]
Maximum frontal breadth	10	116*	122.9	[Rm]
Spinoalveolar height	48(1)	21.5	18.7 ± 0.1	[Rr]
Nasal breadth	54	32*	25.1 ± 0.6	[Rr]
Palatal breadth at C	**	28*	27.6 ± 1.2	[Rr]
Palatal breadth at P4	**	38*	38.6 ± 1.3	[Rr]

M = measurement number and definition by Martin [67] or Bräuer [68]; [Rr] = measurement taken from multiple reference-based virtual reconstruction; [Rm] = measurement taken from virtual reconstruction based on preserved morphology. Measurements are in mm.

* Measurement estimated by Vlček as unilateral half of the current measurement, therefore, multiplied by two for the comparison purpose.

** Defined by Vlček [18].

### 4.2 Analysis of morphological affinity

Cranial measurements were taken on the reconstruction of the Zlatý kůň cranium to analyse the morphological affinity of the skull (Table 3, S4 Table). To filter out the effect of general size, morphological variation was inspected using LDA on log-shape ratios derived from 13 cranial measurements (see Table 4) corresponding to measurements in the Howells database.

**Table 3:** Raw measurements for the Zlatý kůň cranium.

M	Variable	Zlatý Kůň
1	Maximum cranial length	197.7
5	Length of the skull base	99.2
8	Maximum cranial breadth	137.2
9	Minimum frontal breadth	97.2
10	Maximum frontal breadth	122.9
17	Basibregmatic height	128.5
20	Auriculo-bregmatic height	109.5
23	Horizontal circumference	566.3
26	Frontal sagittal arc	140.0
27	Parietal sagittal arc	124.8
28	Occipital sagittal arc	125.9
40	Basion-prosthion length	96.3
45	Bi-zygomatic breadth	125.1
48	Naso-alveolar height	61.8
51	Orbital breadth	43.1
52	Orbital height	30.0
54	Nasal breadth	25.1
55	Nasal height	43.5
61	Maxillo-alveolar breadth	62.0
63	Internal palate breadth	39.2

M = measurement number and definition by Martin [67] or Bräuer [68].

**Table 4:** Function loadings of population DFA for size-adjusted craniometric variables.

Measurement		Function 1	Function 2
Martin [67]	Howells [69]		
M1	GOL	16.96	-1.84
M5	BNL	-5.11	2.46
M8	XCB	-14.63	-10.82
M10	XFB	-1.36	18.95
M17	BBH	6.68	-14.34
M40	BPL	4.45	1.58
M45	ZYB	11.88	-5.02
M48	NPH	-0.01	-0.83
M51	OBB	13.13	4.83
M52	OBH	-10.54	-6.09
M54	NLB	-1.04	4.97
M55	NLH	4.78	3.49
M61	MAB	-4.01	-3.73

**Fig 8:**
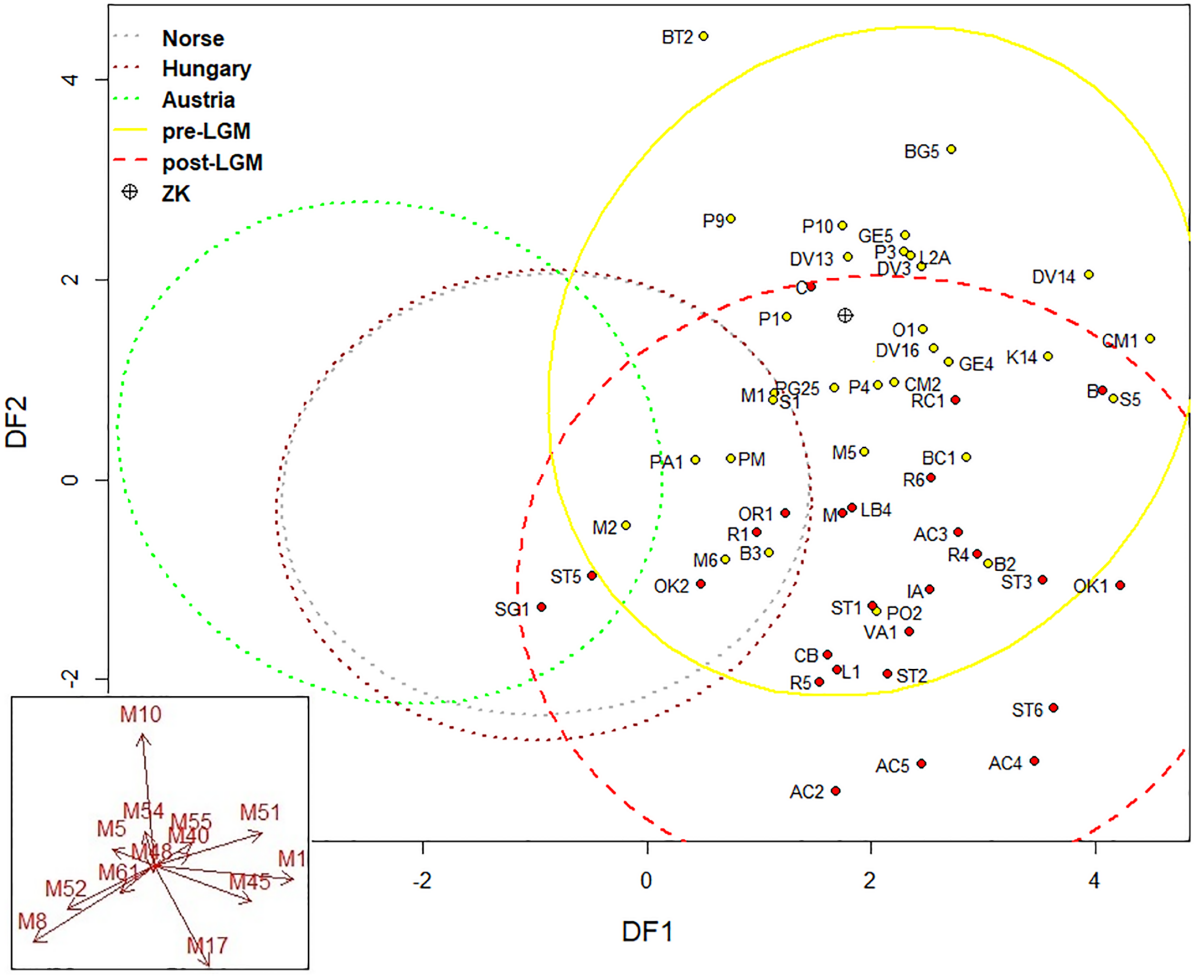
Linear discriminant analysis of recent and fossil samples. Variability of individual samples is indicated by 95% confidence ellipses. Norse, Hungary, Austria = recent samples (individual points not shown for better legibility); ZK = Zlatý kůň. Specimen acronyms are in S3 Table. Red arrows indicate loadings of log-shape variables on the discriminant axes.

Four discriminant functions were obtained, but only the first two were important for our study. The first discriminant axis separated past UP populations from the recent European populations (Fig 8). The second discriminant axis separated the pre-LGM and post-LGM samples. The coefficients of the discriminant functions revealed that the first function differentiated maximum cranial length, maximum cranial breadth, orbital breadth, bi-zygomatic breadth and orbital height. The second discriminant function differentiated maximum frontal breadth, basibregmatic height and maximum cranial breadth (Table 4 and Fig 8). Zlatý kůň was located in the centre of the pre-LGM variation and at the edge of the post-LGM sample variation (Fig 8).

Using cross-validations, 60–70% of individuals were classified into correct population samples whereas the majority of misclassifications occurred either within the variation range of recent populations or of UP ones (Table 5). The Zlatý kůň cranium was classified as belonging to the pre-LGM sample with the probability of 0.96 (Table 5).

**Table 5:** Cross-validation probabilities of classification into population samples from LDA (with absolute numbers) and classification results for Zlatý kůň.

	Austria	Norse	Hungary	pre-LGM	post-LGM
Austria	0.72 (79)	0.17 (19)	0.09 (10)	0.01 (1)	0.00 (0)
Norse	0.05 (5)	0.65 (72)	0.25 (28)	0.03 (3)	0.02 (2)
Hungary	0.12 (12)	0.20 (20)	0.60 (59)	0.05 (5)	0.02 (2)
pre-LGM	0.00 (0)	0.03 (1)	0.10 (3)	0.71 (22)	0.16 (5)
post-LGM	0.00 (0)	0.04 (1)	0.08 (2)	0.19 (5)	0.69 (18)
ZK posterior probability	0.00	0.02	0.00	0.96	0.02

### 4.3 Visual sex assessment

The Zlatý kůň skull was evaluated by a method based on European recommendations [61] as follows: glabella (0), mastoid process (−2), nuchal crest (+1), zygomatic process (0), superciliary arch (+1), frontal eminence (−2), external occipital protuberance (+1), zygomatic bone (−1), frontal inclination (+1). The mandible did not possess a marked level of sexualization and all three characters were neutral (0).

Based on Walker [63], the character scoring was as follows: glabella (3), mastoid process (1), nuchal crest (4), supraorbital margin (2) and mental eminence (3). In addition to Walker’s five features, zygomatic extension was evaluated (3) by the method of Langley et al. [64]. Mandibular flexure was evaluated as slightly flexed, which corresponds to the female sex.

The results of sex assessment based on visual traits are summarized in Table 6. Most of the methods used lean towards the female sex, but methods providing posterior probability show low reliability. In addition, the greatest posterior probability results (over 80%) point towards the male sex.

**Table 6:** Summary of visual methods of sex assessment.

Method	Anatomical region	Number of variables	Posterior probability	Sex assessment
Ferembach et al. (1980)	Cranium	9	-	F
Ferembach et al. (1980)	Mandible	4	-	?
Ferembach et al. (1980)	Skull	13	-	F
Loth and Henneberg (1996)	Mandible	1	-	F
Walker (2008)	Skull	3	0.59	F
Walker (2008)	Skull	2	0.78	F
Walker (2008)	Skull	2	0.84	M
Walker (2008)	Skull	2	0.75	F
Walker (2008)	Skull	2	0.82	M
Walker (2008)	Skull	3	0.73	F
Langley et al. (2017)	Skull	3	0.73	F

### 4.4 Morphometric sex assessment

Raw cranial measurements were used in the sex classification of Zlatý kůň based on an UP sample of reliably sexed specimens. The random forest algorithm was used to order variables according to their importance in the sex classification and only the most important variables were used to create a classification model with LDA. In order not to overfit the model with too many variables, a subset of variables with the greatest cross-validation success rate was chosen. As a result, four variables (bi-zygomatic breadth, naso-alveolar height, horizontal circumference and orbital breadth, in decreasing order; Table 7) were included in the classification model, which correctly classified 94% of individuals with the posterior probability threshold of 0.5 (Table 8). In 50% of individuals, all of which were classified correctly, the posterior probability was greater than 0.95. When this model was applied to Zlatý kůň, the cranium was classified as belonging to a female with a probability of 0.98 (Table 8).

**Table 7:** Importance for first 10 variables in random forest for sex.

Variable	Importance
M45	1.883
M48	1.262
M23	1.075
M51	0.906
M8	0.680
M1	0.499
M26	0.454
M20	0.411
M55	0.318
M5	0.286

**Table 8:** Cross-validation probabilities of classification into sex groups from LDA (with absolute values between parentheses) and classification results for Zlatý kůň.

	0.5 threshold		0.95 threshold		
	F	M	F	M	I
F	1.0 (13)	0.0 (0)	0.54 (7)	0.0 (0)	0.46 (6)
M	0.1 (2)	0.9 (17)	0.0 (0)	0.53 (10)	0.47 (9)
ZK posterior probability	0.98	0.02	-	-	-

F (females), M (males), I (indeterminate = posterior probability lower than 0.95).

**Fig 9:**
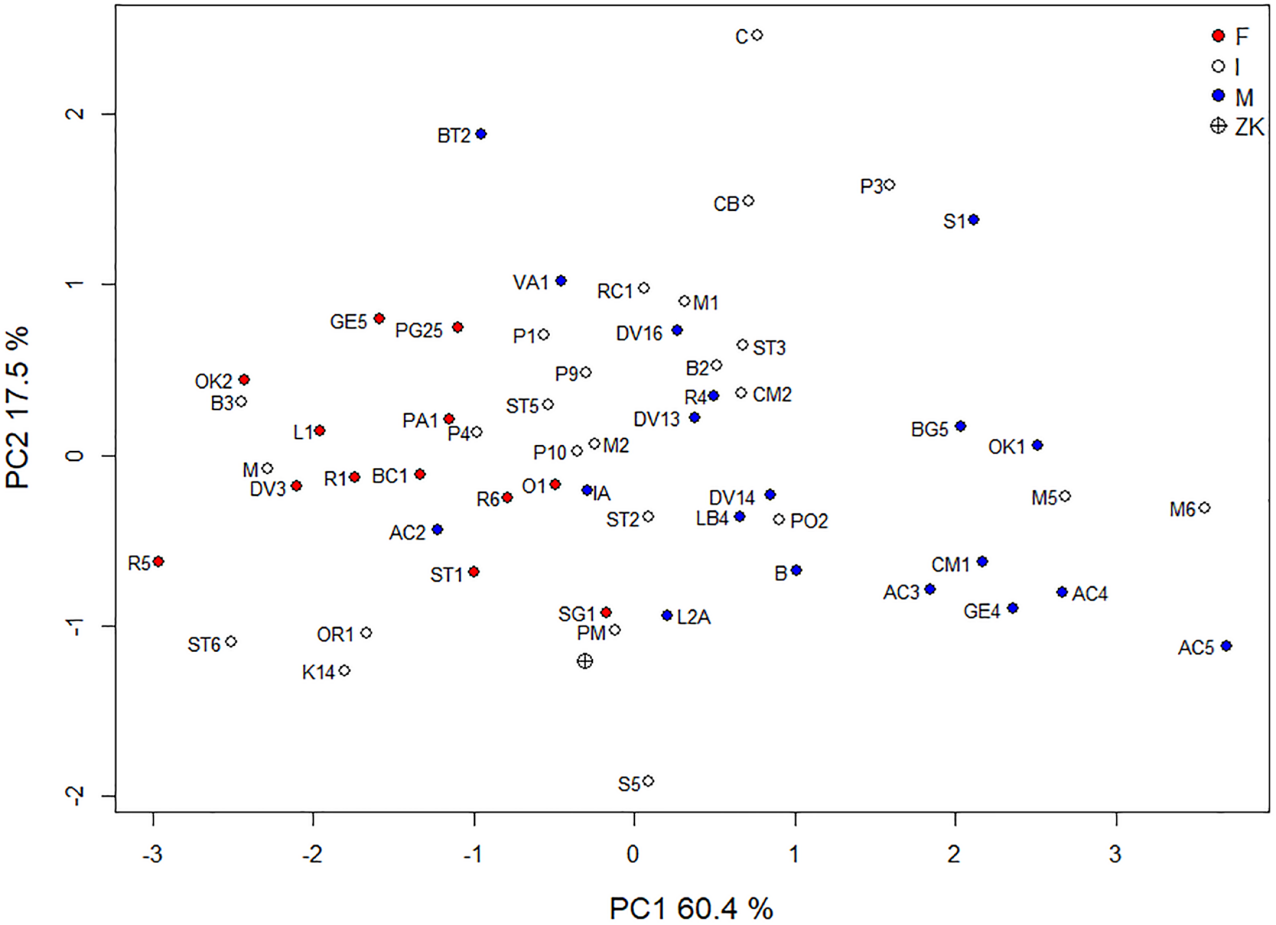
Principal component analysis performed on the four most important cranial variables for sex classification. Specimen acronyms are in S3 Table.

To visually present the classification results, PCA was performed on this subset of variables. Clear clusters of males and females were obtained, separated along the first principal component axis, which accounted for 60% of the total variation (Fig 9). This shows that the combination of the four variables is important for sex classification based on skulls of UP specimens. However, the Zlatý kůň cranium is plotted very close to the transition between males and females (Fig 9).

## 5 Discussion

Preservation is a limiting factor in palaeoanthropological studies, as fossils are often found in a fragmentary and incomplete state. Similarly, the Zlatý kůň individual is represented mainly by fragmentary cranial remains with large missing regions. Until now the Zlatý kůň skull has been included almost exclusively in studies analysing the expression of single cranial or mandibular traits in Late Pleistocene hominins [19,28,39,41,42,86]. More widely focused analyses considered only the well preserved mandible [87] or a restricted part of the frontal bone [40]. The reconstruction of the Zlatý kůň cranium facilitates its inclusion into a wider range of morphometric analyses.

### 5.1 Reconstruction

The present reconstruction is based on virtual computer techniques which offer more objective tools than manual methods based on the anatomical knowledge of an experienced anthropologist. Using virtual tools, the mandible was safely separated from the rest of the cranium and all fragments, except for the maxilla, were re-assembled following anatomical rules. Although an approach employing dental occlusion could also be taken into consideration [88], it would not be appropriate in this situation for the two following reasons: (a) the heavy attrition of the teeth and (b) the extraction of the teeth and their subsequent placement back in their alveoli. Both circumstances prevent a reliable occlusal match between the maxilla and the mandible.

Every reconstruction depends on prior assumptions [57] and the method used to estimate missing parts. Both effects should be considered before and during subsequent analyses. The main assumption in the reconstruction of the Zlatý kůň cranium was the use of a recent sample of modern human crania to estimate missing parts. However, keeping in mind our further aim to analyse morphological affinity and sex of the Zlatý kůň individual, the contemporaneous cranium of Moča [9] was included and the reference sample was divided into several subsamples, which potentially could have affected the reconstruction. The use of either the recent reference sample or the contemporaneous reference specimen resulted in a negligible effect on the final reconstruction, as did the use of either the male or the female subsample.

Missing regions of the Zlatý kůň cranium were located mainly in the middle face, the temporal fossa and the cranial base. The preserved and missing areas influence the efficacy of the TPS estimation. The TPS algorithm is known to work well on smooth surfaces such as the cranial vault [82], but fortunately, the missing regions in the area of the face of Zlatý kůň were not large and were well surrounded by structures with preserved morphology. Also, a large number of semilandmarks ensured an adequate warping of the reference morphology. However, it must be noted that some areas with high variability and low integration, such as the cranial base, may be predicted with very low accuracy [89].

### 5.2 Sex attribution

Reconstructions of the biology and behaviour of past populations rely on the accurate assessment of the sex of fossil specimens [90–92]. For many years, skull robusticity had been commonly used as an indicator of sex [62,93]. However, sexing of fossil individuals requires specific methods because the degree of cranial sexual dimorphism changed over time [90].

The sex assessment based on visual cranial traits, using the methods of Ferembach et al. [61] and Loth and Henneberg [65], identified the Zlatý kůň individual as a female. Discriminant functions designed by Walker [63], combining scores of visually rated characters, did not provide a clear conclusion; DFA combining the glabellar area and mental eminence and another DFA involving the orbital margin and mental eminence indicated male sex because the scores of both characters were equal to 3. Such a score value was earlier found only in 1.9% of females for the orbital margin, 31% of males and females for the glabella and in 17% of females for the mental eminence in the Euro-American population [63]. Therefore it is not surprising that these two DFAs indicated that the Zlatý kůň individual was a male. According to the hyperfeminine value for the mastoid process (1), the last four DFAs indicated female sex. Mental eminence, included in the DFAs indicating the male sex of the Zlatý kůň individual, exhibits a particularly high scoring error [94]. Despite only slight intra- and inter-observer disagreement in Walker’s other visual traits, subjectivity in trait scoring leads to differences in sex classification [94].

Simultaneously, a new classification technique called decision tree was used for assessing sex on the basis of three non-metric features [64]. The posterior probability of 0.73 indicated female sex of the Zlatý kůň skull. As emphasized by Garvin and Klales [95], the decision tree model produces a strong sex bias, with females correctly classified in 94% and males only in 49% of the test sample.

Results obtained by visual sexing techniques cannot be considered reliable because classification methods of sex estimation from the skull are population-specific. It should be remembered that the equations used here were devised from a recent human population sample, which may not be an appropriate model for sexing an Upper Palaeolithic individual. What is known about the extent and character of sex-related variation within the UP population is based on only a small sample [90]; it is generally accepted that fossil specimens showed higher robustness than today’s human population. This fact is described as an evolutionary trend of skeletal gracilization which goes hand in hand with craniofacial feminization, manifested particularly in males but not excluding females [90,96]. It is therefore necessary to anticipate a certain degree of shifts in the outcomes of sex estimations: A fossil skull classified as an uncertain female by a method derived from a contemporary reference sample might more likely turn out to be a female in the UP context.

One of the most daunting situations in sex assessment is when conflicting results are obtained from the same skeleton [97]. Therefore, the risk of misclassification must be reduced as much as possible, which is why we applied a more objective method based on cranial measurements. However, population specificity of cranial measurements [98] affects sex estimation methods [99]. To overcome this problem, we used an Upper Palaeolithic sample of individuals whose sex was estimated by reliable techniques. Most of the selected dimensions were from the facial skeleton and are traditionally used in methods of sex estimation from the skull [61]. Using the morphometric approach to sex estimation produced evidence that the Zlatý kůň individual was more likely a female, with a high posterior probability (0.98). Estimates with posterior probability over 95% can be considered reliable [100]. However, despite the good performance in the UP sample, we have to keep in mind the small size of the reference sample and the possibility of an error.

### 5.3 Cranial morphology in the UP context

The Zlatý kůň cranium exhibits a clear morphological affinity to specimens dated to the period prior the Last Glacial Maximum. This fact is in concordance with earlier descriptions of the archaic morphology of the Zlatý kůň specimen (see section 2.2) but contrasts with direct radiocarbon dating, which estimated the age of the specimen at ca 15.4 ky cal BP [21]. Chronologically, Zlatý kůň belongs to the period after the LGM (23 to 19 ky cal BP [1]) but before the onset of a sudden warming phase which started 14.7 ky cal BP (Bølling) and is related to the main reoccupation of Central Europe [2].

Craniometrics is one of the main tools of biological distance analysis [101] reflecting similarities between human groups [102]. Since the publication of comparative studies on the craniometrics and genetic variation of different human populations of the world, it is believed that cranial measurements reflect genetic variation and neutral evolution [103,104]. Craniometric variation is thus geographically structured [105] and it is widely used for population history [106] or population affinity analyses [107], even in palaeoanthropological studies [108,109]. Morphological similarity means closer relatedness, which may be caused by common ancestry, gene flow or a combination of factors [110]. However, biological affinity of a cranium separated temporally from the reference population is hard to interpret because, instead of a putative population continuity, it could be a result of selective pressure or genetic drift [103]. To explore possible interpretations of the position of the Zlatý kůň specimen, we need to consider palaeodemographic processes in the Late Glacial period and the sources of their evidence.

The LGM is known to have had a profound effect on the biogeography of many animal and plant species [111,112] as well as on human populations in the Upper Palaeolithic, which resulted in population movements across the continent [3]. Traditionally, the environmental deterioration during the LGM has been linked to an abandonment of Central and Northern Europe. The process of subsequent recolonization has been extensively studied mainly since the radiocarbon technique provided a chronological dating of the localities concerned [22]. Based on these data, a two-phase colonization model for Northern Europe was proposed, with an initial pioneer phase and a later residential phase of settlement, coinciding with the onset of Bølling 13 ky uncal BP [22]. However, this theory was questioned on the basis of data calibration which converts radiocarbon dates to calendar ages [113,114]. Calibrated dates showed that increased human occupation correlates with climatic amelioration but they did not permit the inference about two separate phases [2,113].

Further evidence of the recolonization process in the Late Glacial period is provided by genetic studies of present-day populations. The Franco-Cantabrian refuge was confirmed to be a major source of the European gene pool [115]. The process of recolonization from a refuge is documented by the distribution of mitochondrial haplogroups H and V, which spread throughout Europe during the expansion of humans from the southwest to the northeast in the Late Glacial period [115,116]. However, a contemporary population can only provide information conserved within it and cannot say anything about processes of abandonment or unsuccessful colonization. Such processes can be inferred only from archaeological material, which, however, always provides only partial knowledge and needs to be confronted with various sources of information.

Craniometric data obtained from UP specimens indicate a major shift in cranial variability corresponding to the LGM [27]. The results have been ascribed to neutral demographic processes even though they were mainly influenced by facial measurements (nasal height, nasal width, orbital height and least frontal breadth), which are also highly environmentally plastic [117], and it is therefore possible that a portion of the cranial variation was influenced by the changing climate. The analysis could not tell whether the change occurred already in the refuge or during the recolonization process [27]. Concordant results were obtained from palaeogenetic data [29,30]. The pre-LGM population most probably survived the LGM but was quickly replaced around 14.5 ky cal BP [29]. Finally, the disappearance of certain haplogroups from the European gene pool also points to possible local extinctions [29,30].

Processes of abandonment or unsuccessful colonization were given less attention in the context of Late Glacial biogeography, but they are equally important because they reflect limits and constraints on human adaptation [3]. A growing body of evidence shows that Central Europe was not completely abandoned during the LGM [6,13,118,119]. Human populations were certainly reduced, but they were still possibly present throughout the Pleniglacial [23,120]. New findings from eastern Central Europe point to a possible existence of at least seasonally occupied areas with a milder environment [118,121,122] where some vertebrate species survived, possibly attracting human hunters. This indicates the importance of local microclimatic conditions [118] and suggests that the processes taking place in eastern Central Europe were not necessarily the same as in the more extensively studied western part of Central Europe [3,123]. Following a large-scale synthesis of spatiotemporal processes documented by evidence of raw material gathering, production technologies and calibrated ^14^C dates, it seems that the eastern and western populations were not completely separated by the LGM. Instead, they maintained contact through the LGM, as supported by similarities between the western Badegoulian and eastern early Epigravettian cultures [4]. The late-glacial recolonization of Central Europe was then realized from two source areas in the west (Franco-Cantabria) and in the east (Carpathian basin), which is described as the so-called bidirectional model [4,124].

Based on this knowledge of population processes, the biological affinity of the Zlatý kůň specimen can be interpreted in several different ways. Given the complexity of population processes and new evidence of a certain continuity of settlement in Central Europe, the biological affinity of the Zlatý kůň cranium may reflect actual population affinity to the pre-LGM population sample. This would be supported by the fact that the main variables separating the pre- and post-LGM samples are not located in the facial skeleton, which is more sensitive to selective pressure [117]. Secondly, migration from a refuge into a new region with a different kind of selective pressure could have led to this phenotype. The majority of Central European specimens from the pre-LGM period (Dolní Věstonice, Předmostí and Mladeč) are also very close to the Zlatý kůň skull, which supports both interpretations. However, other post-LGM specimens from Central Europe (of which there are very few) are located well within the post-LGM population (Fig 8), which does not lend support to the idea of a selective pressure leading to the same phenotype. Although Brewster et al. [27] showed that the LGM had a disruptive effect on craniometric variation even in specimens from a common region, they did not test for geographic differences due to the dispersed nature of the data. Finally, the position of Zlatý kůň within the range of craniometric variation could be caused simply by genetic drift, which plays an important role especially in small populations. That is probably not far from the post-glacial reality.

We offer possible interpretations based on two assumptions: (1) Cranial variation reflects biological similarity; and (2) Zlatý kůň has been dated to ca 15.4 ky cal BP. Although the Zlatý kůň cranium is referred to as a Magdalenian specimen, the associated artefacts do not show any strong cultural diagnostic features. In addition, the radiocarbon dating was performed on a fragment ‘most probably from the cranial base of the buried individual’ [21]. The choice of the dated material is thus archaeologically suspect [22]. On the other hand, it is still the only evidence of the age of the Zlatý kůň specimen which is supported by the presence of reliably dated Magdalenian sites in the close proximity of the Zlatý kůň site and by the absence of any important Early Upper Palaeolithic site in the whole of the Bohemian Karst [21]. The results obtained by our craniometric analysis possibly reflect Late Glacial processes only discernible from archaeological material. However, in the case of Zlatý kůň, we cannot rule out the possibility that the results indicate a real population affinity.

### 5.4 Future research directions

Our virtual reconstruction of the Zlatý kůň cranium allowed us to scrutinize the previous sex attribution and to explore its biological affinity. The estimation of the sex of an individual from the skull is strongly population-specific. As we discuss in the previous section, the population affinity of the Zlatý kůň individual should be tested further using other methods. A repeated, reliable dating of the fossil, properly performed in all aspects of the radiocarbon technique, will be key [22]. Next, a genetic analysis could provide a reliable sex determination. And last but not least, genetic and isotopic analyses could contribute to the mosaic of information about population processes in the Upper Palaeolithic [125].

## 6 Conclusion

The present study reports on a virtual reconstruction of the incomplete cranium from the Zlatý kůň site in the Bohemian Karst, Czech Republic. The reconstruction allowed us to acquire important cranial measurements and explore the sex attribution and morphological affinity of the specimen. The sex of the individual could not be reliably assessed using visual methods, as those have been developed on a human recent population sample. A metric approach based on reliably sexed Upper Palaeolithic specimens confirmed the previously suggested female sex of the individual with high probability. However, the small size of the reference sample should be kept in mind.

Despite the shift in craniometric variation caused by the Last Glacial Maximum, Zlatý kůň exhibits morphological affinity with the pre-LGM population. This can be explained by various phenomena arising from the complex population history in the Late Glacial period. However, a new dating would allow us to verify the chronological attribution of the fossil. If the Zlatý kůň specimen is really from the Late Glacial period, further investigations and analyses of ancient DNA could contribute to the mosaic of information about population processes in Central Europe.

## Supporting information

S1 Table List of landmarks. (PDF)

S2 Table List of anatomical landmarks and semilandmarks used in different steps of reconstruction. (PDF)

S3 Table Upper Paleolithic comparative sample. (PDF)

S4 Table Descriptive statistics of cranial measurements for UP sample with values for Zlatý kůň. (PDF)

S1 File Video illustration of the skull reconstruction. (AVI)

S2 File Supplementary bibliography. (PDF)
