# Supplementary material for: Virtual reconstruction of the Upper Palaeolithic skull from Zlatý Kůň, Czech Republic: Sex assessment and morphological affinity
Source: PLoS One. 2018 Aug 30;13(8):e0201431. doi: 10.1371/journal.pone.0201431 (PMC6116938; doi:10.1371/journal.pone.0201431)
Supplement: S1 Table — (PDF) [file pone.0201431.s001.pdf]

S1 Table
Landmarks used in the reconstruction of Zlatý kůň cranium

S1 Table: List of landmarks

Landmark name	Abbreviation	Definition
Cranium		
Akanthion	ak	Bräuer, 1988
Apertion	apt	Guyomarc'h et al., 2014
Articular eminence	ae	Guyomarc'h, Brůžek, 2010
Asterion	ast	Bräuer, 1988
Basion	ba	Bräuer, 1988
Bregma	br	Bräuer, 1988
Canine alveolare externus	cae	Guyomarc'h, 2011
Ectomolare	ecm	Bräuer, 1988
Frontomalare orbitale	fmo	Bräuer, 1988
Frontomalare temporale	fmt	Bräuer, 1988
Glabella	g	Bräuer, 1988
Glenoidale laterale	gll	The most lateral point of the glenoid fossa.
Inion	i	Bräuer, 1988
Jugale	ju	Bräuer, 1988
Lambda	la	Bräuer, 1988
Lateromastoideale	lm	Guyomarc'h, 2011
Mastoideale	ms	Bräuer, 1988
Nariale	nr	Guyomarc'h, Brůžek, 2010
Nasion	n	Bräuer, 1988
Nasomaxillofrontale	nmf	Guyomarc'h et al., 2014
Nasospinale superius	nss	Bräuer, 1988
Opisthion	o	Bräuer, 1988
Opisthocranion	op	Bräuer, 1988
Orbitale	or	Bräuer, 1988
Porion	po	Bräuer, 1988
Prosthion	pr	Bräuer, 1988
Rhinion	rhi	Bräuer, 1988
Stephanion	st	Bräuer, 1988
Tuber articulare	tua	Guyomarc'h, Brůžek, 2010
Zygomaticofaciale*	zf	The inferior point of the zygomaticofacial foramen.
Zygomaticotemporale*	zt	The inferior point of the zygomaticotemporal foramen.
Zygomaxillare	zm	Guyomarc'h et al., 2014
Zygoorbitale*	zo	Bräuer, 1988
Mandible*		
Genion	Mge	Bräuer, 1988
Gnathion	Mgn	Bräuer, 1988
Infradentale	Mid	Bräuer, 1988
Kondylion anterius	Mkda	The most anterior point on the condylar process.
Kondylion laterale	Mkdl	Bräuer, 1988
Kondylion mediale	Mkdm	Bräuer, 1988
Linguale	Mlin	Bräuer, 1988
Pogonion	Mpg	Bräuer, 1988

* landmarks digitised only on Zlatý kůň (not on the reference sample crania)
