# Supplementary material for: Virtual reconstruction of the Upper Palaeolithic skull from Zlatý Kůň, Czech Republic: Sex assessment and morphological affinity
Source: PLoS One. 2018 Aug 30;13(8):e0201431. doi: 10.1371/journal.pone.0201431 (PMC6116938; doi:10.1371/journal.pone.0201431)
Supplement: S2 Table — (PDF) [file pone.0201431.s002.pdf]

S2 Table: List of anatomical landmarks and semilandmarks used in different steps of reconstruction.

Abbreviation	Midplane estimation	Calvarium reconstruction	Right zygomatic reconstruction	Maxilla alignment	Cranium reconstruction
Cranium					
ak					*m
aptL					*
aptR				*	*
aeL					*
aeR			*		*
astL		*m			*
astR					*
ba					*m
br	*	*			*
caeL					*
caeR				*	*
ecmL					*
ecmR				*	*
fmoL		*m			*
fmoR			*		*
fmtL		*m			*
fmtR			*		*
g	*	*			*
gllL		*m			*
gllR					*
i	*	*			*
juL					*
juR			*		*
la	*	*			*
lmL		*m			*
lmR					*
msL		*m			*
msR					*
nrL					*
nrR				*	*
n	*	*			*
nmfL		*m			*
nmfR					*
nss				*	*
o					*m
op	*	*			*
orL					*
orR			*m		*
poL		*m			*
poR					*
pr					*m
rhi					*m
stL		*m			*
stR					*
tuaL		*m			*
tuaR					*

Abbreviation	Midplane estimation	Calvarium reconstruction	Right zygomatic reconstruction	Maxilla alignment	Cranium reconstruction
zfR			*m		
ztR			*		
zmL					*m
zmR					*m
zoR			*m		
Mandible					
Mge	*				
Mgn	*				
Mid	*				
MkdaL		*			
MkdaR		*			
MkdlL		*			
MkdlR		*			
MkdmL		*			
MkdmR		*			
Mlin	*				
Mpg	*				
**Total: Anatomical landmarks**	cranium 6; mandible 5	22 (10)	8 (3)	5	48 (7)
**Total: Curve semilandmarks**	0	16 (0)	7 (2)	7	152 (54)
**Total: Surface semilandmarks**	0	374 (144)	20 (7)	30	358 (104)

Asterisk (*) marks that the landmark was used in the reconstruction step; (m) means that the landmark was missing.
