# Supplementary material for: Virtual reconstruction of the Upper Palaeolithic skull from Zlatý Kůň, Czech Republic: Sex assessment and morphological affinity
Source: PLoS One. 2018 Aug 30;13(8):e0201431. doi: 10.1371/journal.pone.0201431 (PMC6116938; doi:10.1371/journal.pone.0201431)
Supplement: S3 Table — (PDF) [file pone.0201431.s003.pdf]

S3 Table: Upper Paleolithic comparative sample

Individual	Abbr.	LGM	Country	Sex	Sex-based	Reference-sex	Reference-cranial measurements
Arene Candide 2	AC2	post	Italy	M	pelvis	Tarsi et al. 2006; Villotte 2009	Paoli et al. 1980; Sergi et al. 1974
Arene Candide 3	AC3	post	Italy	M	pelvis	Tarsi et al. 2006; Villotte 2009	Paoli et al. 1980; Sergi et al. 1974
Arene Candide 4	AC4	post	Italy	M	pelvis	Tarsi et al. 2006; Villotte 2009	Paoli et al. 1980; Sergi et al. 1974
Arene Candide 5	AC5	post	Italy	M	pelvis	Tarsi et al. 2006; Villotte 2009	Paoli et al. 1980; Sergi et al. 1974
Baousso da Torre 2	BT2	pre	Italy	M	pelvis	Villotte et al. 2017	Villotte et al. 2017
Barma del Caviglione 1	BC1	pre	Italy	F	pelvis	de Lumley et al. 2016	de Lumley et al. 2016; Guipert et al. 2014
Barma Grande 1	BG1	pre	Italy	M	pelvis	de Lumley et al. 2016; Villotte et al. 2011	De Lumley et al. 2016; Graziosi 1942
Barma Grande 5	BG5	pre	Italy	M	pelvis	de Lumley et al. 2016; Villotte et al. 2011	De Lumley et al. 2016; Graziosi 1942
Brno 2	B2	pre	Czech	I	?	Matiegka 1934 (M)	Matiegka 1934; Coppola 2013
Brno 3	B3	pre	Czech	I	?	Matiegka 1934 (F)	Matiegka 1934; Coppola 2013
Cap Blanc	CB	post	France	I	skull/PC	von Bonin 1935 (F)/Villotte et al. 2015 (F)	von Bonin 1935
Cro-Magnon 1	CM1	pre	France	M	pelvis	Gambier et al. 2006	Vallois, Billy 1965
Cro-Magnon 2	CM2	pre	France	I	pelvis	Gambier et al. 2006 (I)	Vallois, Billy 1965
Cro-Magnon 3	CM3	pre	France	I	pelvis	Gambier et al. 2006 (I)	Vallois, Billy 1965
Cussac L2A	L2A	pre	France	M	pelvis,skull	Villotte et al. 2015; Guyomarc´h et al. 2017	Guyomarc´h et al. 2017
Dolní Věstonice 13	DV13	pre	Czech	M	pelvis, DNA	Trinkaus and Svoboda 2006; Fu et al. 2016; Mittnik et al. 2016	Sládek et al. 2000
Dolní Věstonice 14	DV14	pre	Czech	M	pelvis, DNA	Trinkaus and Svoboda 2006; Fu et al. 2016; Mittnik et al. 2016	Sládek et al. 2000
Dolní Věstonice 15	DV15	pre	Czech	M	pelvis, DNA	Trinkaus and Svoboda 2006; Fu et al. 2016; Mittnik et al. 2016	Sládek et al. 2000
Dolní Věstonice 16	DV16	pre	Czech	M	pelvis, DNA	Trinkaus and Svoboda 2006; Fu et al. 2016	Sládek et al. 2000
Dolní Věstonice 3	DV3	pre	Czech	F	pelvis	Trinkaus and Svoboda 2006	Sládek et al. 2000; Manolis et al. 1996; Jelínek 1964

Individual	Abbr.	LGM	Country	Sex	Sex-based	Reference-sex	Reference-cranial measurements
Grotte des Enfants 4	GE4	pre	Italy	M	pelvis, skull	Villotte 2009	Verneau 1902; Henke 1989
Grotte des Enfants 5	GE5	pre	Italy	F	pelvis	Villotte 2009	Verneau 1902; Coppola 2013
Chancelade	C	post	France	I	PC	Villotte 2009 (F)	pers.obs.
Iboussiere A	IA	post	France	M	pelvis	pers.obs.	pers.obs.
Kostenki 1	K1	post	Russia	I	-	-	Henke 1989
Kostenki Markina Gora 14	K14	pre	Russia	I	pelvis,skull	Rogachev 1957 (M); Howells 1973, https://web.utk.edu/~auerbach/ (F)	Jelínek 1964; Henke 1989; Howells, 1973, https://web.utk.edu/~auerbach/
Lafaye 1	L1	post	France	F	pelvis	Villotte 2009	pers.obs.
Laugerie-Basse 4	LB4	post	France	M	pelvis	Villotte 2009	pers.obs.
Le Bichon	B	post	Switzerland	M	pelvis	Villotte 2009	Chauviere 2008
Le Peyrat 5	LP5	post	France	M	pelvis	Samsel et al. 2016	Samsel et al. 2016
Maritza 2	MA	post	Italy	I	PC	Villotte 2009	Henke 1989; Borgognini-Tarli 1972
Mladec 1	M1	pre	Czech	I	skull	Szombathy 1925 (M); Wolpoff 2006 (F)	Wolpoff 2006; Matiegka 1934
Mladec 2	M2	pre	Czech	I	skull	Henke 1989 (F); Wolpoff 2006 (F)	Wolpoff 2006; Henke 1989; Mallegni et al. 1999
Mladec 5	M5	pre	Czech	I	skull	Frayer et al. 2006 (M)	Frayer et al. 2006
Mladec 6	M6	pre	Czech	I	skull	Frayer et al. 2006 (M)	Frayer et al. 2006
Moca	M	post	Slovakia	I	skull	Šefčáková et al. 2011 (F)	Šefčáková et al. 2011
Oberkassel 1	OK1	post	Germany	M	pelvis	Trinkaus 2015	pers.obs.; Bonnet 1919
Oberkassel 2	OK2	post	Germany	F	pelvis	Trinkaus 2015	pers.obs.; Bonnet 1919
Ortucchio1	OR1	post	Italy	I	-	-	Henke 1989; Parenti 1960
Ostuni 1	O1	pre	Italy	F	pelvis, DNA	Villotte 2009; Fu et al. 2016	Coppola 2013
Paglicci 25	PG25	pre	Italy	F	pelvis	Villotte 2009	Mallegni et al. 1999
Pataud 1	PA1	pre	France	F	pelvis	Villotte et al. 2015	Billy 1975
Pavlov 1	PV1	pre	Czech	M	pelvis, DNA	Trinkaus and Svoboda 2006; Fu et al. 2016	Sládek et al. 2000
Pestera cu Oase 2	PO2	pre	Romania	I	-	-	Crevecoeur et al. 2009; Trinkaus et al. 2003
Pestera Muierii 1	PM	pre	Romania	I	skull	Dobos et al. 2010 (I)	Dobos et al. 2010
Predmosti 1	P1	pre	Czech	I	skull	Matiegka 1934 (M)	Matiegka 1934
Predmosti 10	P10	pre	Czech	I	skull	Matiegka 1934 (F)	Matiegka 1934; Velemínská, Brůžek 2008

Individual	Abbr.	LGM	Country	Sex	Sex-based	Reference-sex	Reference-cranial measurements
Predmosti 3	P3	pre	Czech	I	skull	Matiegka 1934 (M)	Matiegka 1934; Manolis et al. 1996
Predmosti 4	P4	pre	Czech	I	skull	Matiegka 1934 (F)	Matiegka 1934; Velemínská, Brůžek 2008
Predmosti 9	P9	pre	Czech	I	skull	Matiegka 1934 (M)	Matiegka 1934; Manolis et al. 1996
Rochereil 1	RC1	post	France	I	skull	Ferembach 1974 (M)	pers.obs.
Romito 1	R1	post	Italy	F	second. diag.	Villotte 2009; Villotte et al. 2015	Mallegni,Fabbri 1995
Romito 3	R3	post	Italy	M	pelvis	Villotte 2009	Mallegni,Fabbri 1995
Romito 4	R4	post	Italy	M	second. diag.	Villotte 2009; Villotte et al. 2015	Mallegni,Fabbri 1995
Romito 5	R5	post	Italy	F	second. diag.	Villotte et al. 2015	Mallegni,Fabbri 1995
Romito 6	R6	post	Italy	F	pelvis	Villotte 2009	Mallegni,Fabbri 1995
San Teodoro 1	ST1	post	Italy	F	pelvis	Villotte 2009	pers.obs.
San Teodoro 2	ST2	post	Italy	I	skull	D'Amore et al. 2009 (M)	Henke 1989; Parenti 1960
San Teodoro 3	ST3	post	Italy	I	pelvis,second. diag./skull	Villotte 2009 (I)/D'Amore et al. 2009 (M)	Graziosi 1947
San Teodoro 5	ST5	post	Italy	I	pelvis,PC/ skull	Villotte 2009 (I)/D'Amore et al. 2009 (M)	Graziosi 1947; Henke 1989
San Teodoro 6	ST6	post	Italy	I	skull	D'Amore et al. 2009 (F)	Graziosi 1947
San Teodoro 7	ST7	post	Italy	I	skull	D'Amore et al. 2009 (M)	Graziosi 1947
St Germain la Riviere	SG1	post	France	F	pelvis	Henry-Gambier et al. 2002	pers.obs.
Sunghir 1	S1	pre	Russia	M	pelvis/DNA	Trinkaus et al. 2014; Villotte 2009/Poltoraus et al. 2000	Trinkaus et al. 2014
Sunghir 5	S5	pre	Russia	I	skull	Trinkaus et al. 2014 (I)	Trinkaus et al. 2014
Vado all Arancio 1	VA1	post	Italy	M	pelvis	Villotte 2009	Pardini et al. 1981
Villabruna 1	V1	post	Italy	M	pelvis, DNA	Vercellotti et al. 2008; Villotte 2009; Fu et al. 2016	Vercellotti et al. 2008
Zlatý kůň	ZK	post	Czech	I	skull	Vlček 1991 (F)	pers.obs.

Abbr. = specimen acronym; LGM: chronology regarding the LGM; Sex (F = female, M = male, I = unknown); Sex-based: sexual diagnosis type.
