# Supplementary material for: Virtual reconstruction of the Upper Palaeolithic skull from Zlatý Kůň, Czech Republic: Sex assessment and morphological affinity
Source: PLoS One. 2018 Aug 30;13(8):e0201431. doi: 10.1371/journal.pone.0201431 (PMC6116938; doi:10.1371/journal.pone.0201431)
Supplement: S4 Table — (PDF) [file pone.0201431.s004.pdf]

S4 Table:

Descriptive statistics of cranial measurements following Martin (1928) and Bräuer (1988) for UP sample with values for Zlatý kůň

Variable	N (F)	N (M)	Mean (F)	SD (F)	Mean (M)	SD (M)	N (pre-LGM)	N (post-LGM)	Mean (pre-LGM)	SD (pre-LGM)	Mean (post-LGM)	SD (post-LGM)	Min	Max	Zlatý kůň
M1	13	24	187.0	7.2	193.5	6.5	35	29	193.0	7.6	189.1	6.9	177.0	205.6	197.7
M5	11	18	100.4	5.6	102.1	14.2	19	24	105.6	13.6	100.3	6.7	87.0	151.0	99.2
M8	13	24	136.0	4.1	140.6	4.7	35	29	140.0	8.1	138.8	4.5	127.0	166.5	137.2
M9	13	23	94.5	4.8	99.0	5.1	33	30	99.9	4.7	95.3	4.3	83.5	112.0	97.2
M10	13	21	115.2	5.2	119.5	6.1	34	27	120.8	5.9	115.0	5.0	104.0	136.9	122.9
M17	12	17	132.8	5.0	134.4	5.6	21	28	133.3	3.3	136.2	6.8	125.0	149.0	128.5
M20	12	19	114.4	4.0	116.6	7.2	27	24	116.3	7.3	114.4	6.6	100.0	136.0	109.5
M23	13	15	521.2	14.7	550.6	17.7	23	25	533.5	19.4	535.7	21.6	496.0	590.0	566.3
M26	10	22	129.8	3.4	135.8	6.8	34	20	133.6	7.9	132.2	8.3	116.0	154.0	140.0
M27	10	21	134.5	8.9	133.4	8.9	35	20	131.4	7.0	135.1	8.8	117.0	159.0	124.8
M28	8	16	121.3	10.6	121.3	7.3	25	19	121.4	6.6	118.5	10.5	105.0	143.0	125.9
M40	7	17	97.7	8.6	97.0	7.3	14	22	101.7	6.5	95.4	6.6	85.0	110.0	96.3
M45	12	17	129.6	5.2	141.9	8.2	22	24	135.2	8.4	137.9	8.4	119.2	155.0	125.1
M48	11	21	64.5	2.0	67.9	3.8	27	27	66.9	4.2	66.4	4.5	58.0	78.0	61.8
M51	11	22	39.7	1.6	43.0	3.2	28	26	41.9	2.4	41.6	3.4	37.0	49.0	43.1
M52	12	23	29.5	2.5	30.0	2.4	29	28	29.9	2.5	30.2	2.4	26.0	37.0	30.0
M54	12	20	24.5	2.0	25.4	2.6	29	26	26.3	1.8	23.9	2.0	20.0	30.0	25.1
M55	12	21	48.5	3.0	50.9	3.6	26	27	51.2	4.0	49.3	3.7	43.0	60.0	43.5
M61	11	18	61.7	4.7	63.8	3.4	25	20	63.5	4.9	62.5	4.5	50.0	72.2	62.0
M63	9	16	36.6	4.0	37.8	2.4	16	19	37.8	4.1	38.0	3.0	32.0	48.0	39.2
