# Supplementary material for: Virtual reconstruction of the Upper Palaeolithic skull from Zlatý Kůň, Czech Republic: Sex assessment and morphological affinity
Source: PLoS One. 2018 Aug 30;13(8):e0201431. doi: 10.1371/journal.pone.0201431 (PMC6116938; doi:10.1371/journal.pone.0201431)
Supplement: S2 File — (PDF) [file pone.0201431.s006.pdf]

Auerbach BM. Howells database online. https://web.utk.edu/~auerbach/ (F).

Billy G. 1975. Etude anthropologique des restes humains de l'Abri Pataud. In: Movius HI., editor. Excavation of the Abri Pataud. Les Eyzies (Dordogne). Cambridge: Harvard University, Peabody Museum, American School of Prehistoric Research. p 201–261.

Bonnet R. 1919. Der diluviale Menschenfund von Oberkassel bei Bonn. In: Verworn M, Bonnet R, Steinmann G, editors. Die Skelet. Weisbaden: Verlag von JF Bergmann. p 11–185.

Borgognini-Tarli SM. 1972. Etude anthropologique d'un squelette mésolithique provenant de la grotte Maritza (Avezzano, Abruzzo). In: Etudes sur le quaternaire dans le monde : Union Internationale pour l'étude du Quaternaire, VIIIe Congrès INQUA, Paris. p 1005–1011.

Bräuer G. 1988. Ostéometrie. In: Knussmann R, editor. Anthropologie. Handbuch der vergleichenden Biologie des Menschen. Begründet von Rudolf Martin. Stuttgart: G. Fischer Verlag. p 160–231.

Coppola D. 2013. Il Riparo di Agnano nel Paleolitico superiore - La sepoltura Ostuni 1 ed i suoi simboli.

Crevecoeur I, Rougier H, Grine F, Froment A. 2009. Modern human cranial diversity in the late pleistocene of Africa and Eurasia: Evidence from Nazlet Khater, Peştera cu Oase, and Hofmeyr. Am J Phys Anthropol 140:347–358.

D'Amore G, Di Marco S, Tartarelli G, Bigazzi R, Sineo L. 2009. Late Pleistocene human evolution in Sicily: comparative morphometric analysis of Grotta di San Teodoro craniofacial remains. J Hum Evol 56:537–550.

De Lumley H. 2016. La Grotte du Cavillon Sous la falaise des Baousse Rousse, Grimaldi, Vintimille, Italie. Paris: CNRS éditions.

Dobos A, Soficaru A, Trinkaus E. 2010. The Prehistory and Paleontology of the Pestera Muierii (Romania). Liège: ERAUL 124.

Ferembach D. 1974. Les hommes de l'Épipaléolithique et du Mésolithique de la France et du Nord-Ouest du Bassin méditerranéen. Bull Mem Soc Anthropol Paris 2:201–236.

Frayer D, Jelínek J, Oliva M, Wolpoff M. 2006. Aurignacian male crania, jaws and teeth from the Mladeč caves, Moravia, Czech Republlic. In: Teschler-Nicola M, editor. Early Modern Humans at the Moravian Gate: The Mladeč Caves and their Remains. Vienna: Springer. p 185–272.

Fu Q, Posth C, Hajdinjak M, Petr M, Mallick S, Fernandes D, Furtwängler A, Haak W, Meyer M, Mittnik A, Nickel B, Peltzer A, Rohland N, Slon V, Talamo S, Lazaridis I, Lipson M, Mathieson I, Schiffels S, Skoglund P, Derevianko AP, Drozdov N, Slavinsky V, Tsybankov A, Cremonesi RG, Mallegni F, Gély B, Vacca E, Morales MRG, Straus LG, Neugebauer-Maresch C, Teschler-Nicola M, Constantin S, Moldovan OT, Benazzi S, Peresani M, Coppola D, Lari M, Ricci S, Ronchitelli A, Valentin F, Thevenet C,

Wehrberger K, Grigorescu D, Rougier H, Crevecoeur I, Flas D, Semal P, Mannino MA, Cupillard C, Bocherens H, Conard NJ, Harvati K, Moiseyev V, Drucker DG, Svoboda J, Richards MP, Caramelli D, Pinhasi R, Kelso J, Patterson N, Krause J, Pääbo S, Reich D. 2016. The genetic history of Ice Age Europe. Nature 534:200–205.

Gambier D, Bruzek J, Schmitt A, Houët F, Murail P. 2006. Révision du sexe et de l'âge au décès des fossiles de Cro-Magnon (Dordogne, France) à partir de l'os coxal. Comptes Rendus Palevol 5:735–741.

Graziosi P. 1942. L'uomo fossile della Barma Grande ai Balzi Rossi nel Museo di Mentone. Arch per l'Antropologia e l'Etnologia 72:22–51.

Graziosi P. 1947. Gli uomini paleolitici della Grotta di San Teodoro (Messina). Riv di Sci Preist 2:123–233.

Guipert G, de Lumley H, de Lumley M-A. 2014. Reconstruction du crâne Barma del Caviglione 1 (Dame du Cavillon), Baoussé-Roussé, Grottes de Grimaldi Gaspard. Ann dell'Università di Ferrara 10:239–244.

Guyomarc'h P, Bruzek J. 2010. Dimorphisme sexuel du crâne de sujets identifiés (collection Olivier, MNHN, Paris): Évaluation par morphométrie géométrique. Bull Mem Soc Anthropol Paris 22:216–229.

Guyomarc'h P. 2011. Reconstitution Faciale par Imagerie 3D : Variabilité morphométrique et mise en oeuvre informatique.

Guyomarc'h P, Dutailly B, Charton J, Santos F, Desbarats P, Coqueugniot H. 2014. Anthropological facial approximation in three dimensions (AFA3D): computer-assisted estimation of the facial morphology using geometric morphometrics. J Forensic Sci 59:1502–1516.

Guyomarc'h P, Samsel M, Courtaud P, Mora P, Dutailly B, Villotte S. 2017. New data on the paleobiology of the Gravettian individual L2A from Cussac cave (Dordogne, France) through a virtual approach. J Archaeol Sci Reports 14:365–373.

Henke W. 1989. Jungpaläolithiker und mesolithiker Beiträge zur Anthropologie.

Henry-Gambier D, Bruzek J, Murail P, Houët F. 2002. Révision du sexe du squelette magdalénien de Saint-Germain-la-Rivière (Gironde, France). Paléo 14:205–212.

Howells W. 1973. Cranial Variation in Man. A Study by Multivariate Analysis of Patterns of Differences among Recent Human Populations. Cambridge: Harvard University Press.

Chauvière F-X. 2008. La grotte du Bichon: un site préhistorique des montagnes neuchâteloises. Neuchâtel Off musée Cant d'archéologie Neuchâtel.

Jelínek J. 1964. Betrachtungen uber die Verwandtschaft der anthropologischen Funde Dolni Vestonice, Abri Pataud und Markina Gora.

Mallegni F, Bertoldi F, Manolis SK. 1999. The Gravettian female human skeleton from Grotta Paglicci, south Italy. Homo 50:127–148.

Mallegni F, Fabbri PF. 1995. The human skeletal remains from the upper palaeolithic burials found in Romito cave (Papasidero, Cosenza, Italy). Bull Mem Soc Anthropol Paris 7:99–137.

Manolis SK, Mallegni F. 1996. The Gravettian fossil hominids of Italy. Anthropologie 34:99–108.

Matiegka J. 1934. Homo předmostensis, fosilný člověk z Předmostí na Moravě.

Mittnik A, Wang C-C, Svoboda J, Krause J. 2016. A molecular approach to the sexing of the triple burial at the Upper Paleolithic site of Dolní Věstonice. PLoS One 11:e0163019.

Paoli G, Parenti R, Sergi S. 1980. Gli scheletri mesolitici della caverna delle Arene Candide (Liguria). Mem dell'Istituto Ital di Paleontol Um Roma 3:33–154.

Pardini E, Lombardi Pardini EC. 1981. I Paleolitici di Vado all'Arancio (Grosseto). Arch per l'Antropologia e la Etnol Firenze 61:75–119.

Parenti R. 1960. Calvario cromagnonoide trovato in un deposito mesolitico del bacino fucense (Abruzzo). Arch per l'Antropologia e l'Etnologia 90:5–92.

Poltoraus AB, Kulikov EE, Lebedeva IA. 2000. The molecular analysis of DNA from the remains of three individuals from the Sunghir site (preliminary data). In: Alexeeva TI, Bader N., Munchaev RM, Buzhilova A., Kozlovskaya MV, Mednikova M., editors. Homo Sungirensis, Upper Palaeolithic Man: Ecological and Evolutionary Aspects of the Investigation. Moscow: Scientific World. p 302–314.

Rogachev A. 1957. Mnogosloinye stoyanki Kostenkovsko-Borshevskogo raiona na Donu i problema razvitiya kul'tury v epokhy verkhnego paleolita na Russkoi Ravnine. Mater I Issled Po Arkheologii, SSSR 59:9–134.</

Samsel M, Knüsel CJ, Villotte S. 2016. Réévaluation du sexe et de l'âge au décès du sujet azilien Le Peyrat 5, Saint-Rabier (Dordogne, France). Bull Mem Soc Anthropol Paris 28:213–220.

Sergi S, Parenti R, Paoli G. 1974. Il giovane paleolitico della caverna delle Arene Candide. Mem dell'Istituto Ital di Paleontol Um 2:13–38.

Sládek V, Trinkaus E, Hillson S, Holliday T. 2000. The People of the Pavlovian. Skeletal Catalogue and Osteometrics of the Gravettian Fossil Hominids from Dolni Vestonice and Pavlov. Brno: Academy of Sciences of the Czech Republic, Institute of Archaeology.

Szombathy J. 1925. Die diluvialen Menschenreste aus der Fürst-Johanns-Höhle bei Lautsch in Mähren. Die Eiszeit 2:1-34-95.

Šefčáková A, Katina S, Mizera I, Halouzka R, Barta P, Thurzo M. 2011. A Late Upper Palaeolithic skull from Moča (The Slovak Republic) in the context of Central Europe. Acta Musei Natl Pragae 67:3–24.

Tarsi T, Noto F, Martínez-Labarga C, Giampaolo R, Babalini C, Scano G, Contini I, Lorente JA, Lorente M, Pacciani E, Silvestrini M, Del Lucchese A, Maggi R, Lattanzi E, Formicola V, Mallegni F, Martini F, Rickards O. 2006. Ricostruzione della storia genetica per via materna delle comunità paleolitiche dei Balzi Rossi, delle Arene Candide e del Romito, e di quelle neolitiche ed eneolitiche di Samari e di Fontenoce di Recanati. In: Martini AF, editor. La cultura del morire nelle società preistoriche e protostoriche italiane dal paleolitico all'età del rame, origines, progetti, III. Firenze: Istituto Italiano di Preistoria e Prostoria. p 315–346.

Trinkaus E, Svoboda J. 2006. Early Modern Human Evolution in Central Europe: the People of Dolní Věstonice and Pavlov.

Trinkaus E. 2015. The appendicular skeletal remains of Oberkassel 1 and 2. In: Giemsch L, Schmitz RW, editors. The Late Glacial Burial from Oberkassel Revisited. Darmstadt: Verlag Philipp von Zabern. p 75–132.

Trinkaus E, Milota Ş, Rodrigo R, Mircea G, Moldovan O. 2003. Early modern human cranial remains from the Peştera cu Oase, Romania. J Hum Evol 45:245–253.

Trinkaus ME, Buzhilova PA, Mednikova BM, Dobrovolskaya VM. 2014. The People of Sunghir: Burials, Bodies and Behavior in the Earlier Upper Paleolithic. New York: Oxford University Press.

Vallois H, Billy G. 1965. Nouvelles recherches sur les hommes fossiles de l'abri de Cro-Magnon. Anthropologie 69:47–74.

Velemínská J, Brůžek J, Velemínský P, Bigoni L, Šefčáková A, Katina S. 2008. Variability of the Upper Palaeolithic skulls from Předmostí near Přerov (Czech Republic): Craniometric comparison with recent human standards. HOMO- J Comp Hum Biol 59:1–26.

Vercellotti G, Alclati G, Richards MP, Formlcola V. 2008. The Late Upper Paleolithic skeleton Villabruna 1 (Italy): A source of data on biology and behavior of a 14.000 year-old hunter. J Anthropol Sci 86:143–163.

Verneau R. 1902. Les fouilles du prince de Monaco aux Baoussé-Roussé. Un nouveau type humain. Anthropologie 13:561–585.

Villotte S. 2009. Enthésopathies et activités des Hommes préhistoriques - Recherche méthodologique et application aux fossiles européens du Paléolithique supérieur et du Mésolithique.

Villotte S, Brůžek J, Henry-Gambier D. 2011. Révision de l'âge au décès et du sexe des sujets adultes gravettiens. Mémoire LII la Société préhistorique française:209–216.

Villotte S, Churchill SE, Dutour OJ, Henry-Gambier D. 2010. Subsistence activities and the sexual division of labor in the European Upper Paleolithic and Mesolithic: Evidence from upper limb enthesopathies. J Hum Evol 59:35–43.

Villotte S, Samsel M, Sparacello V. 2017. The paleobiology of two adult skeletons from Baousso da Torre (Bausu da Ture) (Liguria, Italy): Implications for Gravettian lifestyle. Comptes Rendus Palevol 16:462–473.

Villotte S, Santos F, Courtaud P. 2015. Brief communication: In situ study of the Gravettian individual from Cussac cave, locus 2 (Dordogne, France). Am J Phys Anthropol 158:759–768.

Vlček E. 1991. L'homme fossile en Europe centrale. Anthropologie 95:409–472.

von Bonin G. 1935. The Magdalenian skeleton from Cap-Blanc in the Field Museum of Natural History. Urbana: University of Illinois.

Wolpoff MH, Frayer DW, Jelínek J. 2006. Aurignacian female crania and teeth from the Mladeč caves, Moravia, Czech Republic. In: Teschler-Nicola M, editor. Early Modern Humans at the Moravian Gate: The Mladeč Caves and their Remains. Vienna: Springer Vienna. p 273–340.
